# Herpes simplex virus type I glycoprotein L evades host antiviral innate immunity by abrogating the nuclear translocation of phosphorylated NF-κB sub-unit p65

**DOI:** 10.3389/fmicb.2023.1178249

**Published:** 2023-05-09

**Authors:** Zhaolong Li, Zhihua Feng, Zhou Fang, Jianghua Chen, Wengzhi Chen, Wangwang Liang, Qi Chen

**Affiliations:** ^1^Institute of Animal Husbandry and Veterinary Medicine, Fujian Academy of Agricultural Sciences, Fuzhou, Fujian Province, China; ^2^Fujian Key Laboratory of Innate Immune Biology, College of Life Sciences, Fujian Normal University Qishan Campus, Fuzhou, Fujian Province, China

**Keywords:** HSV-1, GL, NF-κB, p65, evasion

## Abstract

Nuclear factor (NF)-κB plays an important role in the innate immune response by inducing antiviral genes’ expression. However, the herpes simplex virus 1 (HSV-1) virus has developed multiple ways to interfere with NF-κB activity to escape the host antiviral response. Here, we found that HSV-1 envelope glycoprotein L(gL) markedly inhibits interferon (IFN) production and its downstream antiviral genes. Our results showed that ectopic expression of gL inhibited IFN-β promoter activation, and decreased IFN-β production, the expression of IFN-stimulated genes (ISGs), and inhibited immunologic stimulant (poly I:C) induced activation of IFN signaling pathway. Depletion of gL by short interfering RNA (siRNA) significantly upregulated IFN-β and ISG production. Further study showed that the N-terminus of the gL bound to the Rel homology domain (RHD) of the p65 and concealed the nuclear localization signal of p65, thereby impeding the translocation of phosphorylated p65 to the nucleus. In summary, our findings indicated that the N-terminal of HSV-1 gL contributes to immune invasion by inhibiting the nuclear translocation of p65.

## Introduction

The innate immune system is the first line of defense against foreign pathogens (Maclean, 1998; Kleinman and Vamvakas, 2010; Wylie et al., 2012). Nuclear factor (NF)-κB plays a major role in the innate immune response by inducing antiviral genes, such as interferon (IFN) and IFN-stimulated genes (ISGs; Medzhitov, 1997). Classical activation of the NF-κB pathway requires the concerted regulation of receptors, adaptor proteins, inhibitors of nuclear factor-kappa B kinase subunits (IKKs), an inhibitor of kappa B (IκBα), and the NF-κB sub-units nuclear factor kappa B sub-unit 1 (NF-κB1, also known as p50) and RELA proto-oncogene, NF-κB subunit (RELA, also known as p65; Le Negrate, 2011). When cellular receptors sense external stimuli, they transmit signals to the IKKs via adaptor proteins, resulting in IKK phosphorylation, IκBα degradation, nuclear translocation of p50/p65, and the induction of IFN and ISG expression (Gregory, 2004; Seth et al., 2005).

Herpes simplex virus 1 (HSV-1) belongs to the alphaherpesvirus subfamily, with a large, linear double-stranded DNA encoding over 80 proteins (Davis-Poynter et al., 1994). HSV-1 causes an acute lytic infection and then establishes lifelong latent infection in the trigeminal ganglia. HSV-1 has evolved several regulatory mechanisms to escape from host immune response (Benetti et al., 2003; Dong et al., 2017). Multiple viral proteins of HSV-1, including envelope glycoprotein G serine/threonine-protein kinase US3 (US3) (Wang J. P. et al., 2013; Wang S. et al., 2013; Xu et al., 2017), nuclear protein UL24 (UL24) (Zhang et al., 2013a,b), DNA polymerase processivity subunit (UL42) (Zhang et al., 2013a,b), and ubiquitin E3 ligase ICP0 (ICP0) can bind p50 and p65 and prevent NF-κB from entering the nucleus (Orvedahl et al., 2007). Besides, neurovirulence protein ICP34.5 (Jin et al., 2008), infected cell protein ICP27 (Xing et al., 2012), tegument protein US11 (Zhang et al., 2016), viral protease VP24 (Su et al., 2015), and virion-host-shutoff protein Vhs bind other effectors of the innate immune system to impede the host antiviral response (Samady et al., 2003; Yuan et al., 2018).

HSV-1 viral genome comprises unique long (UL) and unique short (US) that encode different proteins. Previous studies have reported that UL1-UL8 is involved in virus entry cell, DNA replication, DNA encapsidation, or virion morphogenesis. Recent studies have shown that UL1(gL) forms a complex with envelope glycoprotein H (gH) to trigger HSV-1 fusion and cellular entry (Herold et al., 1991; Fuller and Lee, 1992; Peng et al., 1998; Rasmussen et al., 2007; Chowdhury et al., 2012; Gianni et al., 2015; Lau and Crump, 2015) by preventing gH activation until the complex is dissociated by integrins (Hayden and Ghosh, 2011). Few studies have shown that the UL1-UL8 evades the host’s innate immunity. Our preliminary study has indicated that the UL1 (gL) has significantly downregulated the mRNA level of IFN-β (Supplementary Figure 1). However, whether gL affects the host RNA-sensing pathway is unknown. In the present study, we found that gL acted as an important inhibitor of NF-κB-mediated production of IFN-β and downstream antiviral genes.

Moreover, compared with WT, the knockdown gL of HSV-1 could recover immunostimulatory RNA poly(I:C) induced activation of the IFN signaling pathway. Further study showed that gL interacted with p65 and inhibited the nuclear translocation of phosphorylated p65. Besides, stably knockdown of gL increased the nuclear translocation of phosphorylated p65, but WT not. In summary, these findings expand our understanding of evasion of host antiviral innate immune responses by HSV-1.

## Materials and methods

### Cells, viruses, plasmids, and antibodies

HEK293T cells, Vero cells, and L929 cells were obtained from the American Type Culture Collection (United States). All cells were cultured in 10% Dulbecco’s modified Eagle’s medium (DMEM) containing 10% fetal bovine serum (FBS; PAN-Biotech). Commercial reporter plasmids include IFN-β-Luc (Beijing qualityard biotechnology, China) and pRL-TK plasmid (Promega). HSV-1 was purchased from Taizhou Medical College. Antibodies against RIG-I, MAVS, IKKα, IKKβ, TBK1, IκBα, p65, gL, INKIT, and IRF3 were purchased from Abcam. HSV1-mCherry (VP26 site) virus was stocked in our lab. Mouse monoclonal anti-His and anti-Flag antibodies (mAbs) were purchased from Quanshi Gene (Beijing, China).

### Viral amplification and titer determination

HSV-1 was amplified in Vero cells, and its titer was calculated by Karber’s method (Gilles, 1974). Vero cells (5 × 10^5^) were seeded to each well of a 96-well plate in 90 μl of culture medium, and 10 μl of virus solution was added to the first row at a 1:10 dilution. Eight replicate wells were set up for each dilution series, and three independent experiments were performed. Cytopathic changes were observed every 24 h until no lesions appeared in the wells. Log TCID50 d (s-0.5) was the logarithm of the highest dilution, and the log was the difference between the logarithms of the dilution degrees.

### Transfection and poly(I:C) stimulation of infected cells

HEK 293 T cells (1 × 10^5^) and L929 cells (5 × 10^5^) were cultured in 6-well plates for 12 h. Plasmids (2 μg) were mixed with 6 μg polyetherimide (PEI) and shaken for 10 s, incubated for 5–10 min, and then transfected into HEK 293 T cells and L929 cells for 24 or 32 h (no poly(I:C) groups). Then, 208 μl of poly(I:C) mixture (200 μl Opti-MEM, 2 μg/ml poly(I:C) and 6 μg PEI) were added to the culture medium for 8 h, and samples were collected for RNA and protein extraction.

### Detection of antiviral regulatory factors by real-time quantitative (RT-q) PCR

The culture supernatants of HEK 293 T cells and L929 cells were removed after poly(I:C) stimulation, and the cells were washed with phosphate-buffered saline (PBS) and lysed with 1 ml of TRIzol reagent at 25°C for 5 min. The lysates were transferred to 1.5 ml centrifuge tubes, and RNA was extracted according to the manufacturer’s protocol (TAKALA, Japan). Then, 1 μg of RNA was reverse transcribed to cDNA in a reaction mixture including 1 μl of PrimeScript RT Enzyme Mix I, 1 μl RT Primer Mix, 4 μl of 5% PrimeScript Buffer, and 4 μl of RNase-free dH_2_O. Samples were incubated at 37°C for 15 min and 85°C for 15 min to remove genomic DNA, and then reverse transcription was performed. The resultant cDNA was diluted to 50 ng/μl, and 2 μl was combined with 100 ng of SYBR Premix Ex Taq II (Tli RNaseH Plus), 1 μl of ROX Plus, 0.4 μl of primer F1, 0.4 μl of primer R1, and 7.2 μl ddH_2_O, and primer sequences (UL1-UL8, NF-κB, RIG-I, MAVS, IKKα, IKKβ, TBK1, IκBα, IRF3, UL3, UL1(1-77aa), UL1(78-157aa), UL1(158-225aa) and p65) were shown in Tables 1, 2. Cycle conditions involved a predenaturation step at 95°C for 10 min, then 40 cycles of denaturation at 95°C for 30 s, and annealing and extension at 60°C for 30 s. A melting curve was generated from 72 to 90°C, increasing at 0.1°C per cycle.

**Table 1:** Primers for the amplification of UL1-UL8.

Primers	Sequences	Fragment size
HSV-1-UL1-F	TTTAAACTTAAGCTTATGGATTACAAAGACGATGACGATAAGATGGGGATTTTGGGTTG	675 bp
HSV-1-UL1-R	TGGACTAGTGGATCCTTACTTATCGTCATCGTCTTTGTAATCGAGGCGCCGGGAGT	
HSV-1-UL2-F	GGTACCGAGCTCGGAATGGATTACAAAGACGATGACGATAAGCGGGCCTGCAG	1,005 bp
HSV-1-UL2-R	CACTGGACTAGTGGATCACTTATCGTCATCGTCTTTGTAATCAACCGACCAGTCGATG	
HSV-1-UL3-F	GGTACCGAGCTCGGAATGGATTACAAAGACGATGACGATGTTAAACCTCTGGTCTCAT	708 bp
HSV-1-UL3-R	CACTGGACTAGTGGATTACCTTATCGTCATCGTCTTTGTAATCTCGGCCCCCGAG	
HSV-1-UL4-F	GGTACCGAGCTCGGAATGGATTACAAAGACGATGACGATTCCAATCCAC AGACGAC	600 bp
HSV-1-UL4-R	CACTGGACTAGTGGACTAGCTTATCGTCATCGTCTTTGTAATCGACCCCAAAAGTTTGT	
HSV-1-UL5-F HSV-1-UL5-R	GGTACCGAGCTCGGAATGGATTACAAAGACGATGACGATACCGCACCACGCTC GGTTTTGGACTAGACCATCGTCACCGTCTTTGTAATCGACCCCAAAAGCCT GGTACCGAGCTCGGAATGCTTCAAGGACTGACCTTACCGCACCACGCTC	806 bp
HSV-1-UL6-R	CACTGGACTAGTGGATCACTTATCGTCATCGTCTTTGTAATCTCGTCGGCCGTCG	2031 bp
HSV-1-UL7-F	GGTACCGAGCTCGGAATGGATTACAAAGACGATGACGATGCCGCCGCGAC	641 bp
HSV-1-UL7-R	CACTGGACTAGTGGATCACTTATCGTCATCGTCTTTGTAATCACAAAACTGATAAAAC	
HSV-1-UL8-F	GGTACCGAGCTCGGAATGGATTACAAAGACGATGACGATGACACCGCAGATATCGT	2,253 bp
HSV-1-UL8-R	CACTGGACTAGTGGATCACTTATCGTCATCGTCTTTGTAATCGGCAAACAGAAACGAC	

**Table 2:** The primers for qPCR assay.

Primers	Sequence	Segment
Mouse-IFNβ-F	5′-CAGCTCCAAGAAAGGACGAAC-3′	134 bp
Mouse-IFNβ-R	5′-GGCAGTGTAACTCTTCTGCAT-3′	
Mouse-ISG15-F	5′-TCTTTCTGACGCAGACTGTAGA-3′	135 bp
Mouse-ISG15-R	5′-GGGGCTTTAGGCCATACTCC-3′	
Mouse-TBK1-F	5′-GTACGGCACAGAAGAGTACCT-3′	121 bp
Mouse-TBK1-R	5′-ATGGTAGAATGTCACTCCAACAC-3′	
Mouse-IRF3-F	5′-GAGAGCCGAACGAGGTTCAG-3′	147 bp
Mouse-IRF3-R	5′-CTTCCAGGTTGACACGTCCG-3′	
Mouse-RIGI-F	5′-AAGAGCCAGAGTGTCAGAATCT-3′	106 bp
Mouse-RIGI-R	5′-AGCTCCAGTTGGTAATTTCTTGG-3′	
Mouse-MAVS-F	5′-TGGGGTCACAGTATCAGCC-3′	171 bp
Mouse-MAVS-R	5′-ACTGGGACCAATCTGGGAGAA-3′	
Mouse-IKKα-F	5′-AAGGCCATTCACTATTCTGAGGT-3′	147 bp
Mouse-IKKα-R	5′-GTCGTCCATAGGGGCTCTT-3′	
Mouse-IKKβ-F	5′-GACATCGCATCGGCTCTTAGA-3′	228 bp
Mouse-IKKβ-R	5′-AACGGTCACGGTGTACTTCTG-3′	
Mouse-p65-F	5′-ACTGCCGGGATGGCTACTAT-3′	126 bp
Mouse-p65-R	5′-TCTGGATTCGCTGGCTAATGG-3′	
Mouse-p50-F	5′-ATGGCAGACGATGATCCCTAC-3′	111 bp
Mouse-p50-R	5′-TGTTGACAGTGGTATTTCTGGTG-3′	
Mouse-GAPDH-F	5′-TGGCCTTCCGTGTTCCTAC-3′	178 bp
Mouse-GAPDH-R	5′-GAGTTGCTGTTGAAGTCGCA-3′	
Human-IFNβ-F	5′-CCAACAAGTGTCTCCTCCAAAT-3′	191 bp
Human-IFNβ-R	5′-AATCTCCTCAGGGATGTCAAAG-3′	
Human-ISG15-F	5′-CGCAGATCACCCAGAAGATCG-3′	152 bp
Human-ISG15-R	5′-TTCGTCGCATTTGTCCACCA-3′	
Human-RIGI-F	5′-TGTGCTCCTACAGGTTGTGGA-3′	120 bp
Human-RIGI-R	5′-CACTGGGATCTGATTCGCAAAA-3′	
Human-MAVS-F	5′-TTCTAATGCGCTCACCAATCC-3′	92 bp
Human- MAVS-R	5′-CCATGCTAGTAGGCACTTTGGA-3′	
Human-IKKα-F	5′-ATGAAGAAGTTGAACCATGCCA-3′	110 bp
Human-IKKα-R	5′-CCTCCAGAACAGTATTCCATTGC-3′	
Human-IKKβ-F	5′-CTGGCCTTTGAGTGCATCAC-3′	109 bp
Human-IKKβ-R	5′-CGCTAACAACAATGTCCACCT-3′	
Human-p65-F	5′-GTGGGGACTACGACCTGAATG-3′	121 bp
Human-p65-R	5′-GGGGCACGATTGTCAAAGATG-3′	
Human-p50-F	5′-GAAGCACGAATGACAGAGGC-3′	137 bp
Human-p50-R	5′-GCTTGGCGGATTAGCTCTTTT-3′	
Human-GAPDH-F	5′-ACAACTTTGGTATCGTGGAAGG-3′	101 bp
Human-GAPDH-R	5′-GCCATCACGCCACAGTTTC-3′	
Human-TBK1-F	5′-TGCACCCTGATATGTATGAGAGA-3′	125 bp
Human-TBK1-R	5′-AAATGGCAGTGATCCAGTAGC-3′	
Human-IκBα-F	5′-ACCTGGTGTCACTCCTGTTGA-3′	113 bp
Human-IκBα-R	5′-CTGCTGCTGTATCCGGGTG-3′	
UL1(gL)-F	GAAGGCATATTGGGTTAA	110 bp
UL1(gL)-R	TAAAGGGCCAAGCGCGTTTC	
UL3-F	CTTATCGTCATCGTCTTTGTAATC	178 bp
UL3-R	GATTACAAAGACGATGACGAT	

### Construction of INKIT and *RELA*-knockout cells by CRISPR-Cas9 gene editing

Single guide (sg) RNAs corresponding to the sequences of mouse *Inkit*, and human *RELA* was designed using the http://www.e-crisp.org/E-CRISP/designcrispr.html website. Primers to verify knockout were designed using Primer Premier 5 software. Primers and sgRNAs were synthesized by Fuzhou Platinum Biotechnology Company, and their sequences are shown in Table 3. Opti-MEM (200 μl) was added to two 1.5 ml tubes, and 2 μg knockout carrier was added to one, and 6 μg PEI was added to the other. After mixing and centrifugation, the tubes’ contents were combined, mixed, and centrifuged, and added to the L929 cells or HEK 293 T cells after a 5 min incubation at room temperature. After 6 h of transfection, the culture medium was replaced with fresh DMEM containing 10% FBS. On the second day, puromycin (3 μg/ml) was added to select transfected cells. After 3 days of selection, DNA was extracted from a subset of cells and amplified by PCR to verify the effectiveness of CRISPR/Cas9 gene editing, and then the amplified products sent for sequencing, while a set of peaks appears at the position of the sgRNA, indicating that the sgRNA can be edited effectively. Then, the selected cells were diluted 1,000×, and monoclonal colonies were selected. When colonies reached ~80% confluence, the DNA was reextracted, and PCR amplified to verify *Ink*it and p65 knockout.

**Table 3:** The sequences of siRNA targeting to UL1 (gL) and UL3.

Name	Sequences
Control Sense	5′-UUCUUCGAACGUGUCACGUTT-3′
Control Antisense	5′-ACGUGACACGUUCGGAGAATT-3′
gL-siRNA1Sense	5′-UGCUUUGAUAGACGGUAUATT-3′
gL-siRNA1Antisens	5′-AAUAUACCGUCUAUCAAAGCA-3′
gL-siRNA2Sense	5′-GCUUUGAUAGACGGUAUAUTT-3′
gL-siRNA2Antisens	5′-AAAUAUACCGUCUAUCAAAGC-3′
UL3-siRNA3Sense	5′-UUAUCGTCAUCGTCUUUGTAATC-3′
UL3-siRNA3Antisens	5′-UGAUUACAAAGACGATGACGAT-3′

### Western blot analysis

Total protein contents were determined using a BCA protein concentration assay kit, and 60 μg samples were resolved by running on 12% gels by sodium dodecyl sulfate-polyacrylamide gel electrophoresis (SDS-PAGE), at 80 V for 20 min and then 120 V for 60 min. Proteins were then transferred to polyvinylidene difluoride membranes, and membranes were blocked in 5% skim milk at room temperature for 2 h. Membranes were incubated with primary antibodies (Antibodies against RIG-I, MAVS, IKKα, IKKβ, TBK1, IκBα, p65, gL, UL3, His, Flag, INKIT, and IRF3; diluted to 1:1,000 in 5% skim milk) at 4°C for 12 h, washed with Tris-buffered saline containing 0.1% Tween 20 (TBST) 3 times for 10 min each, and incubated with secondary antibodies (diluted to 1:10,000 in TBST) at room temperature for 2 h before additional washing with TBST for 10 min. Membranes were scanned on a two-color infrared laser imager.

### RNA interference of HSV-1 gL

A siRNA against HSV-1 gL and UL3 was designed on the http://crispr.mit.edu website and synthesized by Shanghai Jiama Pharmaceutical Technology Co., Ltd. (The methods of different concentrations siRNA-gL targe for the gL was shown on the Supplementary Figure 2).

In 10 cm culture dishes, 5 × 10^5^ HEK 293 T cells (6 groups) were cultured to ~80% confluence and infected with HSV-1 (MOI of 0.5) for 4 h, then cell media was replaced by DMEM containing 10% FBS (Supplementary Figure 3). The cells were transfected with an empty plasmid, gL siRNA (1 μg), and UL3 siRNA (1 μg) for 16 h or 12 h (needs poly (I:C) groups), and the cells were added the 2 μg/ml poly (I:C) for 8 h, the cells of 6 groups were harvested for qPCR or WB assay.

### Immunoprecipitation

HEK 293 T cells (5 × 10^5^) were plated in 10 cm culture dishes and infected with virus solution (MOI 0.5). After 24 h of culture, the culture medium was discarded, and the cells were washed with PBS to remove the residual culture medium. The cells were then lysed with lysis buffer (Thermo Fisher Scientific™, RIPA Lysis, and Extraction Buffer, the components are 1% NP-40, 0.25% deoxycholate) on ice and centrifuged at 10,000 g for 10 min at 4°C. The supernatants were incubated with antibodies(NF-κB p65 (D14E12) XP^®^ Rabbit mAb #8242(CST), Phospho-NF-κB p65(Ser536) (93H1) Rabbit mAb #3033(CST), MAVS (D5A9E) Rabbit mAb #24930(CST), RIG-I (D14G6) Rabbit mAb #3743(CST), IKKα Antibody #2682(CST), IKKβ (L570) Antibody #2678(CST), IκBα (44D4) Rabbit mAb #4812(CST), GAPDH (DI16H11) XP^®^ Rabbit mAb #5174(CST), IRF-3 (D6I4C) XP^®^ Rabbit mAb #11904(CST), Phospho-IRF-3 (Ser396) (D6O1M) Rabbit mAb #29047(CST), TBK1/NAK (D1B4) Rabbit mAb #3504(CS), Phospho-TBK1/NAK (Ser172) (D52C2) XP^®^ Rabbit mAb #5483(CST), NF-κB1 p105/p50 Antibody #3035(CST)), His and FLAG mAb at 4°C for 12 h. In control cells, the antibody-targeted glyceraldehyde 3-phosphate dehydrogenase. This mixture was then combined with Protein G Sepharose [Dynabeads™ Protein G and Magnet Starter Pack (Thermo Fisher Scientific 10014D)] and incubated for 6 h, and the samples were washed twice in lysis buffer (the components are 50 mM Tris pH7.5, 100 mM NaCl, 1 mM EDTA, 0.5 mM EGTA, 10% Glycerol, 0.5% Triton-100) and centrifuged at 5,000 rpm for 3 min after each wash. A final 5 min centrifugation step at 3,000 rpm at 4°C was performed to collect the beads. The supernatants were reserved, and samples were boiled in a loading buffer at 99°C for 10 min. The beads were washed twice with lysis buffer, resuspended in 20 μl lysis buffer, and the appropriate amount of a loading buffer was added for denaturation at 99°C for 10 min. Samples were resolved on 15% SDS-PAGE gels, and western blotting was used to detect interesting proteins.

### Fluorescence microscopy

For immunofluorescence, HEK 293 T cells (1 × 10^5^) were plated in the imaging dish (NEST, #801002) of a confocal laser scanning microscope (LSM 780 Laser Confocal Microscope, Zeiss, Germany) for 12 h, transfected with DMEM, empty vector, vector + gL, poly(I:C) group, vector +poly(I:C) group, and vector + gL + poly (I:C). for 12 h, cultured in fresh medium for 16 h, and three of the groups were stimulated with 2 μg/ml poly(I:C) for 8 h. HEK 293 T cells be infected HSV1-mCherry virus (MOI of 0.5) for 4 h, and then transfected with gL siRNA for 12 h. HSV-1+ poly(I:C) and HSV-1-gLsiRNA+poly(I:C) groups add 2 μg/ml poly(I:C) for 8 h. The cells were then fixed in 4% paraformaldehyde for 10 min, washed twice with ice-cold PBS, and permeabilized with 0.1% Triton X-100 for 30 min. After washing twice with ice-cold PBS, the cells were blocked in 5% bovine serum albumin for 2 h at room temperature, washed twice with PBS, and incubated with primary antibodies overnight at 4°C. The next day, the cells were washed twice with PBS, incubated with secondary antibodies (Donkey Anti-Rabbit, Thermo Fisher Scientific, 1:500 dilution) at room temperature for 2 h, washed twice with PBS, stored in a wet box with an anti-fluorescence quenching agent (Thermo Fisher Scientific, ProLong TM Gold Antifade Mountant, #P36930) and imaged on a confocal microscope.

### Statistical analysis

Data are represented as mean ± SD when indicated, and Student’s *T*-test was used for all statistical analyses with the GraphPad Prism 8.0.1 software. Differences between groups were considered significant when *p*-value was <0.05. The fluorescence intensity of p-p65 in nucleus were scanned by ImageJ V1.8.0.112.

## Results

### HSV-1 gL inhibits the transcription of IFN-β and ISGs

**Figure 1:**
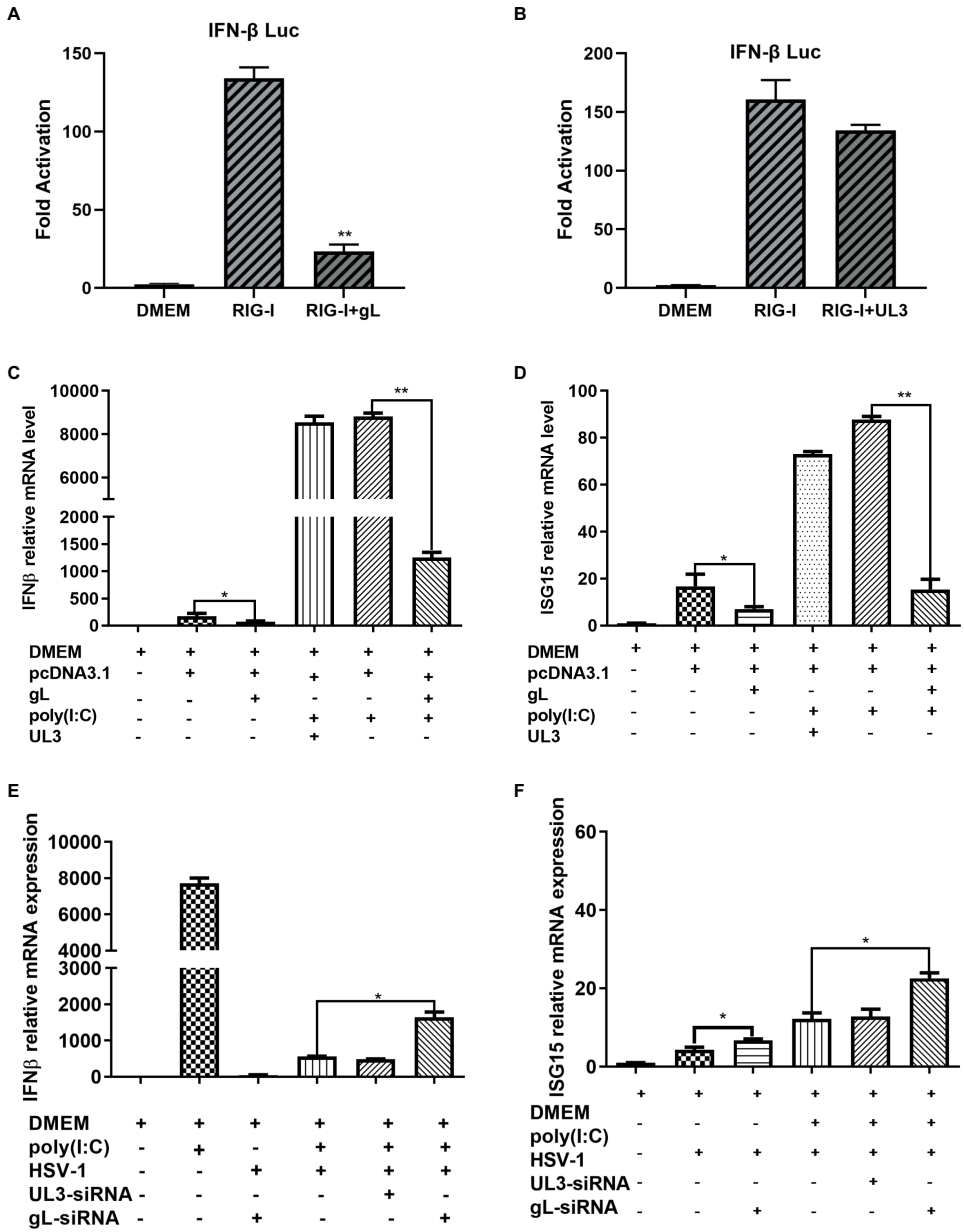
HSV-1 gL inhibits IFN-β activation induced by NF NFκB. **(A-B)** HEK293T cells were cotransfected with IFN-β promoter plasmid (IFN-β-Luc 200 ng), Renilla luciferase (pRL-TK 50 ng) reporter plasmid along with empty vector (200 ng) or, and plasmids encoding cGAS (13 ng) and STING (2.8 ng) or RIG-I (8.6 ng) with gL or UL3 – plasmid (200 ng). Luciferase activity was measured 24 h posttransfection in the cell lysates. **(C)** HEK293T cells were transfected with the different concentrations of pcDNA3.1-gL (400 ng) for 24 h, and then the cells were stimulated with 2 μg/ml poly(I:C) for 8 h, and then harvested and total RNA was extracted for qPCR analysis the mRNA level of IFN-β. HEK 293 T cells were transfected with pcDNA3.1-gL (400 ng), pcDNA3.1 (400 ng), pcDNA3.1-UL3 (400 ng) for 32 h. At 32 h posttransfection, cells were harvested and subjected to qRT-PCR analysis. Next, HEK 293T cells were transfected as described for the preceding panel. At 24 h post-transfection, the cells were transfected with 2 μg/ml poly(I:C) according to the manufacturer’s recommendations for 8 h, the cells were harvested and subjected to qRT-PCR analysis. **(E)** HEK 293 T cells were infected with HSV-1 (MOI of 0.5) for 4 h, then transfected with the different concentrations of gL-siRNA (600 ng) for 20 h, and then the cells were stimulated with 2 μg/ml poly(I:C) for 8 h, and the cells were harvested, and total RNA was extracted for qPCR analysis the mRNA level of IFN-β. HEK 293 T cells were infected with HSV-1 (MOI of 0.5) for 4 h, then transfected with NcsiRNA (1 μg), ULsiRNA (1 μg), or gL-siRNA (1 μg) for 20 h, the cells were harvested and subjected to qRT-PCR analysis. HEK 293 T cells were transfected as described for the preceding panel for 16 h, At 16 h post-transfection, the cell media was replaced by DMEM containing 10% FBS, and the cells were added 2 μg/ml poly(I:C) for 8 h, harvested, and total RNA was extracted for qPCR analysis of the mRNA level of IFN-β and ISG15. The data represent results from one of the triplicate experiments. Error bars represent the standard deviation of three independent experiments. Statistical analysis was performed using Student’s *t*-test with the GraphPad Prism 8.0 software (*p < 0.05; **0.01 < *p* < 0.05).

The NF-κB pathway plays a major role in the innate immune response by inducing antiviral genes such as IFN-β and ISGs. To investigate the role of gL in DNA or RNA sensing signaling on the NF-κB pathway, we first explored whether gL could modulate the cGAS-STING or RIG-I mediated activation of IFN-β promoter. HEK293T cells were cotransfected with IFN-β-Luc reporter plasmid, cGAS and STING or RIG-I expression plasmids along with empty vector or plasmid encoding UL3 or gL. 24 h later, the cells were harvested and subjected to DLR assay. Ectopically expressed gL blocked the activation of IFN-β promoter stimulated by RIG-I shown in Figure 1A. but could not inhibits the cGAS-STING mediated activation of IFN-β promoter (Figure 1B), ectopical expressed UL3 also could not inhibits the RIG-I mediated activation of IFN-β promoter (Figure 1C). Next, we validated the DLR result by IFN-β mRNA levels through quantitative reverse transcription-PCR (qRT-PCR). We performed the assays in HEK 293 T cells transfected with an expressional plasmid for gL. The gL significantly decreased the mRNA of IFN-β and ubiquitin-like modifier (ISG15) in HEK 293 T cells (Figures 1D,E); however, coexpression of HSV-1 UL3, which was applied as a negative control to obviate nonspecific interaction, could not inhibit this transcription of IFN-β and ISG15, Likewise.

Polyinosinic-polycytidylic acid [poly (I:C)], a synthetic viral double-stranded RNA analog, induces innate immune responses in human ovarian granulosa cells and affected endocrine function, and transfection of poly (I:C) into HEK 293 T cells can induce a robust production of IFN-β and ISG15. To determine whether gL could affect the production of IFN-β induced by poly (I:C), HEK 293 T cells were transfected with ectopic gL expressional plasmid for 24 h before poly (I:C) transfection for 8 h. Then cells were harvested and subjected to qRT-PCR to analyze IFN-β and ISG15 mRNA. As shown in Figures 1E,F, ectopic gL expression significantly decreased poly(I:C)-induced transcription of IFN-β and ISG15, but UL3 not. Further study has shown that the down-regulated multiple of IFN-β and ISG15 is inversely proportional to the ectopic expression of gL (Figure 1F).

To further evaluate the role of gL in the inhibition of IFN-β and ISG15 production during HSV-1 infection, HEK 293 T cells were infected with wild type HSV-1 or HSV-1 depleted of gL at an MOI of 0.5. The results have shown that Wild-type HSV-1 significantly reduced the transcription of IFN-β (*p* < 0.05), but gL-depleted HSV-1 significantly upregulated the mRNA levels of IFN-β and ISG15 (Figures 1G,H). After added poly(I:C), gL-depleted HSV-1 similarly increased the mRNA levels of IFN-β and ISG15. Following the increase of siRNA-gL concentration (300–1,200 ng) in the HSV1-gL-siRNA group significantly upregulated the mRNA levels of IFN-β and ISG15 than the only HSV-1 infected group (Figure 1I).

### gL regulates antiviral NF-κB signaling

**Figure 2:**
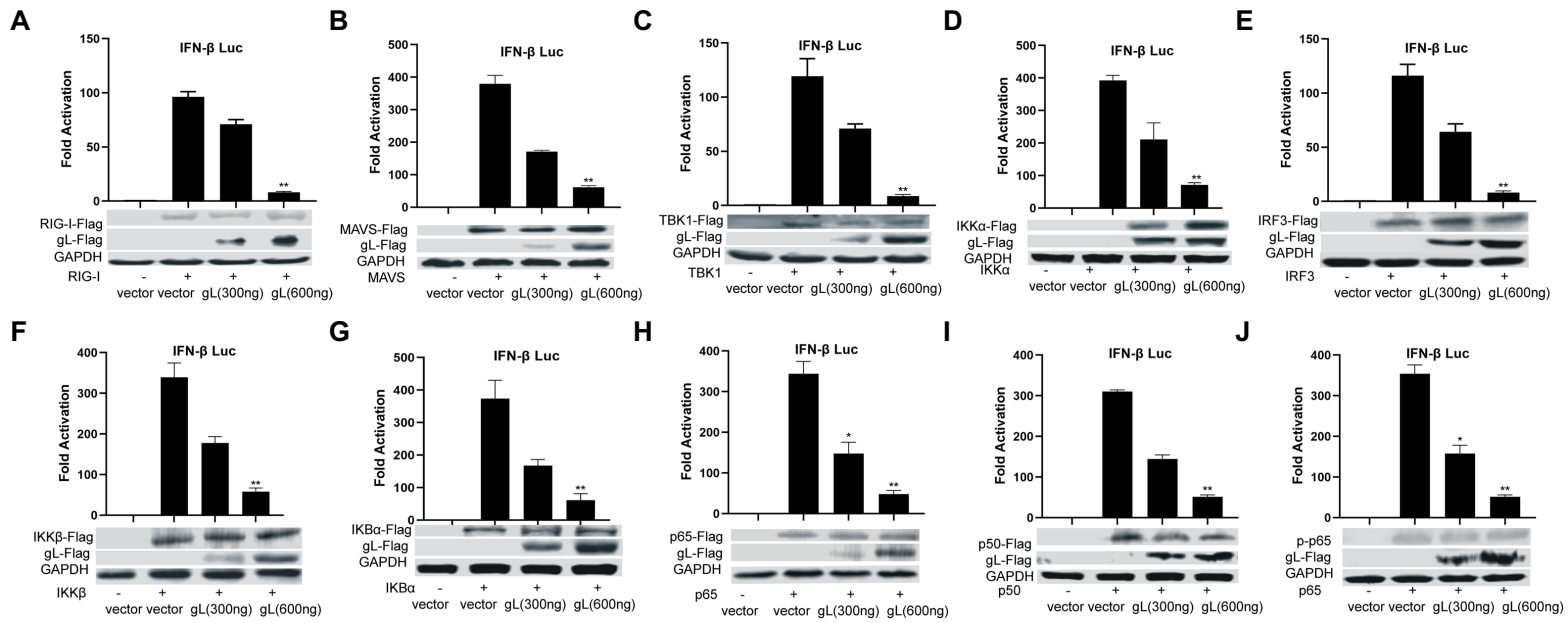
Ectopically expressed gL not downregulated the expression of NF-κB pathway regulatory factors. **(A-J)** HEK293T cells were cotransfected with IFN-β promoter plasmid (IFN-β-Luc 200 ng), Renilla luciferase (pRL-TK 50 ng) reporter plasmid along with empty vector (200 ng) or, and plasmids with gL (300 and 600 ng) or RIG-I, MAVS, IKKα, IKKβ, TBK1, IκBα, IRF3, p50, and p65– plasmid (200 ng) and then the cells were harvested, Luciferase activity was measured 24 h posttransfection in the cell lysates, and then part cells were harvested and subjected to WB analysis with antibodies against inhibitor of NF-κB pathway. The data represent results from one of the triplicate experiments. Error bars represent the standard deviation of three independent experiments. Statistical analysis was performed using Student’s t-test with the GraphPad Prism 8.0 software (*0.01< *p* <0.05 and **0.001< *p* <0.01).

**Figure 3:**
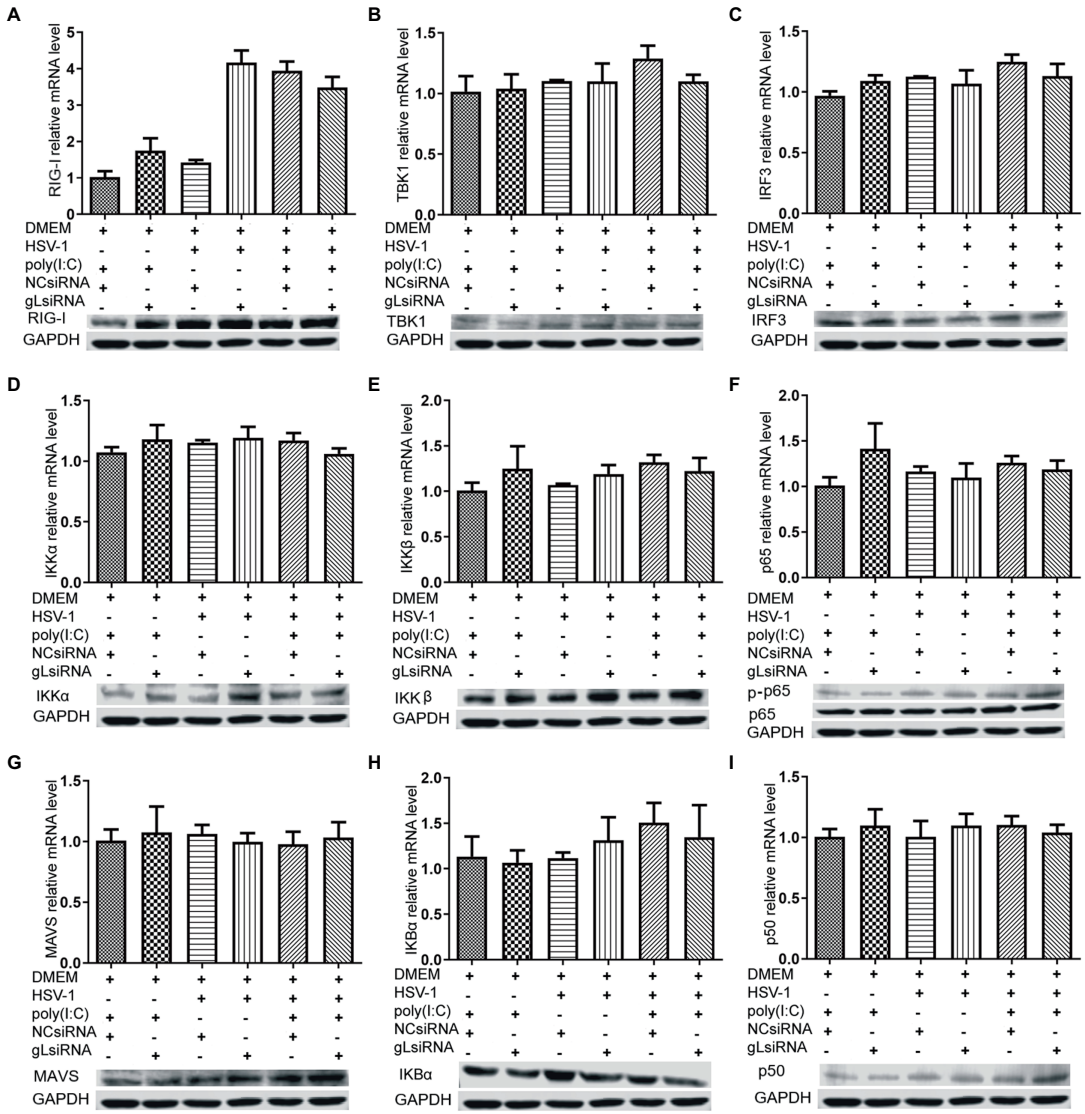
Effects of gL depletion on the expression of NF-κB pathway regulatory factors. **(A-I)** HEK293T cells were infected with HSV-1(MOI of 0.5) for 4h, then transfected with the gL-siRNA (1μg) or NcsiRNA (1μg) for 20h. The cells were harvested and analyzed the mRNA level and protein expression of RIG-I, MAVS, IKKα, IKKβ, TBK1, IκBα, IRF3, p50, and p65. HEK 293T cells were transfected as described for the preceding panel for 16h, and then the cells were stimulated with 2μg/ml poly(I:C) for 8h, and then the cells were harvested, and analysis the mRNA level and protein expression of RIG-I, MAVS, IKKα, IKKβ, TBK1, IκBα, IRF3, p50, and p65. The data represent results from one of the triplicate experiments and the mean±SD from three independent experiments. Statistical analysis was performed using Student’s *t*-test (*0.01< *p* <0.05 and **0.001< *p* <0.01).

NF-κB is a transcription factor complex and promotes cytokines involved in innate immune responses (Negrate et al., 2011). To investigate the role of gL on the NF-κB mediated IFN-β signal pathway, HEK293T cells were transfected with an empty vector or a gL expression plasmid to detect the inhibitors’ interaction with the NF-κB pathway. As shown in Figure 2, the ectopic gL expression did not decrease the mRNA level of NF-κB, nor the mRNA and protein levels of retinoic acid-inducible gene I (RIG-I), mitochondrial antiviral signaling protein (MAVS), a subunit of the inhibitor of nuclear factor-kappa B kinase complex, two inhibitors of nuclear factor-kappa B kinase subunit beta (IKK-β and IKK-a), TANK binding kinase 1 (TBK1), an inhibitor of nuclear factor-kappa B kinase(IκBα), and interferon regulatory factor 3 (IRF3). Besides, ectopic gL expression did not significantly inhibit p65 phosphorylation (Figure 2I), which indicates the active form of NF-κB. Then added the poly(I:C) stimulation, the ectopic gL expression did not significantly affect the mRNA level and protein of NF-κB, RIG-I, MAVS, IKK-β, IKK-a, TBK1, IRF3, IκBα, p65, and p-p65. Similar results were obtained in HEK 293 T cells infected with wild-type or gL-depleted HSV-1 at an MOI of 0.5 under the poly(I:C) stimulated or not (Figure 3). These results suggest that gL does not regulate the levels of NF-κB pathway effectors nor p65 phosphorylation.

Here, the degradation of IκBα has some decreased while the ectopic gL expression without the activated by poly(I:C).IκBα is a negative feedback protein, and if the gL suppresses p65 phosphorylation nuclear translocation, it also will decrease the degradation.

### gL interacts with the NF-κB p65 subunit and inhibits its nuclear translocation

**Figure 4:**
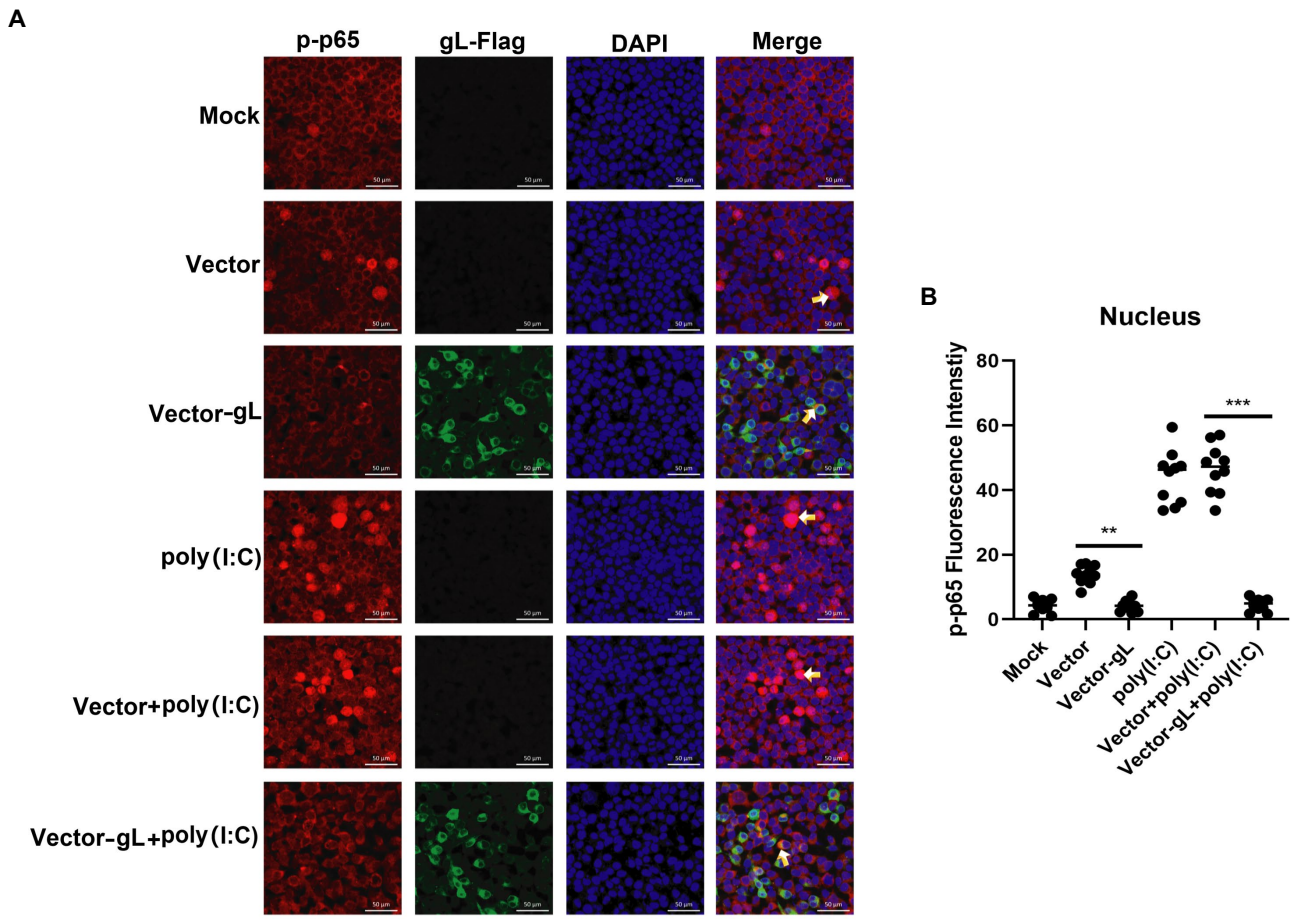
Overexpression of gL inhibits the nuclear translocation of phosphorylated p65. **(A)** HEK293T cells (1 × 10^5^) were plated in the imaging dish of a confocal laser scanning microscope for 12 h, transfected with no plasmid, empty pcDNA3.1, or pcDNA3.1-gL in the presence and absence of poly(I:C) pcDNA3.1-gL plasmid for 12 h, cultured in fresh medium for 12 h, cells of the presence of poly(I:C) group were stimulated with 2 μg/ml poly(I:C) for 8 h, and all groups cells are subjected to confocal analysis the nuclear translocalization of phosphorylated p65. **(B)** The fluorescence intensity of p-p65 in nucleus were scanned by ImageJ. The data represent results from one of the triplicate experiments. Error bars represent the standard deviation of three independent experiments. Statistical analysis was performed using Student’s *t*-test with the GraphPad Prism 8.0 software (**0.001 < *p* < 0.01; ***p < 0.001).

**Figure 5:**
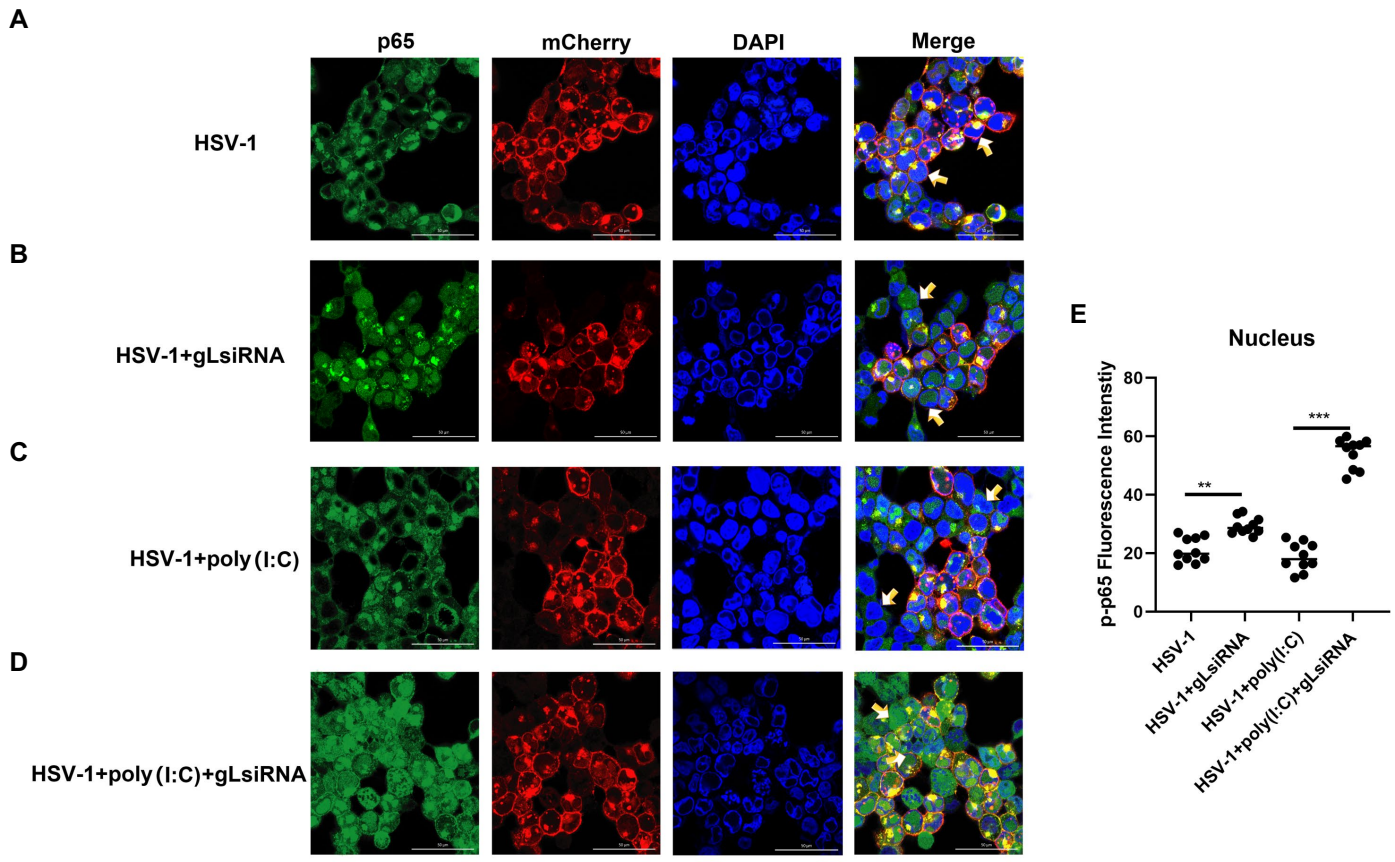
HSV-1 depleted of gL increases the nuclear translocation of phosphorylated p65. **(A)** HEK293T cells of HSV-1 group were infected with mCherry marked HSV-1(MOI of 0.5) for 24 h. **(B)** HEK293T cells were infected with mCherry marked HSV-1(MOI of 0.5) for 16 h, stimulated with 2 μg/ml poly(I:C) for 8 h. **(C)** HEK293T cells were infected with mCherry marked HSV-1(MOI of 0.5) for 4 h, then transfected with 1 μg NCsiRNA for 12, and then stimulated with 2 μg/ml poly(I:C) for 8 h. **(D)** HEK293T cells were infected with mCherry marked HSV-1(MOI of 0.5) for 4 h, then transfected with 1 μg gL-siRNA for 12 h, and then stimulated with 2 μg/ml poly(I:C) for 8 h. **(E)** The fluorescence intensity of p-p65 in nucleus were scanned by ImageJ. The data represent results from one of the triplicate experiments (**0.001 < *p* < 0.01; ***p < 0.001).

The activation of classical NF-κB signaling involves forming the p50/p65 dimer (Seth et al., 2005), its phosphorylation, and its nuclear translocation. Once the dimer enters the nucleus, it initiates the transcription of genes involved in a series of antiviral immune responses. The data as mentioned above led us to hypothesize that gL might act directly on p65 nuclear translocation. To verify this hypothesis, an immunofluorescence assay was performed. HEK 293 T cells were transfected with gL-Flag expression plasmids, the p-p65 nuclear translocation, and the subcellular localization was assessed by confocal microscopy. As shown in Figure 4, the p-p65 nuclear translocation significantly decreased in the gL group than in the control group, and the p-p65 was co-localized with gL (yellow arrow marked). Following poly(I:C)-induced nuclear translocation of phosphorylated p65 was inhibited by gL relative to the control, and the p-p65 was co-localized with gL (Figure 4; yellow arrow marked). Next, we analyzed the potential interaction between the depletion of gL and p-p65 on the HEK 293 T cells infected HSV-1-mCherry (a recombinant virus expressing mCherry). As shown in Figure 5, the depletion of gL increased the nuclear translocation of p65. After added the poly(I:C) stimulation, the depletion of gL more significantly increased the nuclear translocation of p-p65 than the control group.

**Figure 6:**
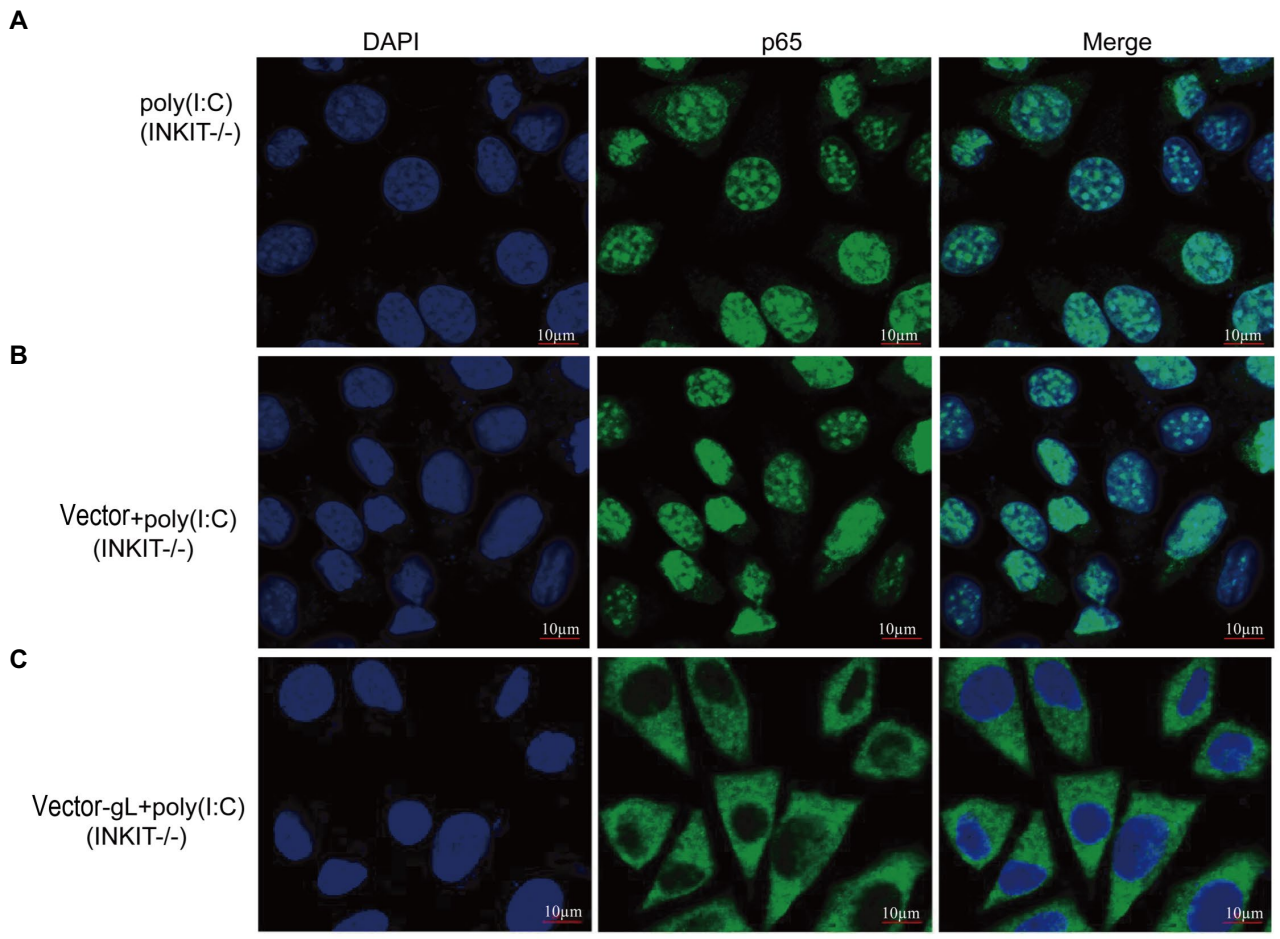
Effects of INKIT on gL-mediated inhibition of p65 nuclear translocation. **(A)** INKIT knockout L929 cells (2 × 10^5^) were plated in the imaging dish of a confocal laser scanning microscope for 24 h, stimulated with 2 μg/ml poly(I:C) for 8 h. **(B,C)** INKIT knockout L929 cells (2 × 10^5^) were plated in the imaging dish of a confocal laser scanning microscope for 12 h, transfected with empty pcDNA3.1, or pcDNA3.1-gL in the presence and absence of poly(I:C) pcDNA3.1-gL plasmid for 12 h, cultured in fresh medium for 12 h, cells were stimulated with or without 2 μg/ml poly(I:C) for 8 h. The data represent results from one of the triplicate experiments. Error bars represent the standard deviation of three independent experiments. Statistical analysis was performed using Student’s *t*-test with the GraphPad Prism 8.0 software.

Inhibitor (INKIT) for NF-κB and IRF3 is associated with IKKα/β and TBK1/IKK and inhibits the recruitment and phosphorylation of p65 and IRF3 (Lu et al., 2017). To further examine whether gL could affect the nuclear translocation of p65 on INKIT, INKIT knockout L929 cells were transfected with ectopic expression of gL prior to the poly(I:C) stimulation and performed immunofluorescence assay. As shown, ectopic expression of gL in INKIT knockout cells did not noticeably affect p65 nuclear translocation (Figure 6), suggesting that gL does not bind INKIT, nor is INKIT involved in gL-mediated regulation of p65 nuclear translocation. Therefore, the result suggested that gL mediated p-p65 nuclear translocation regulation while the IκBα was degraded.

### gL protein interacts with p65

**Figure 7:**
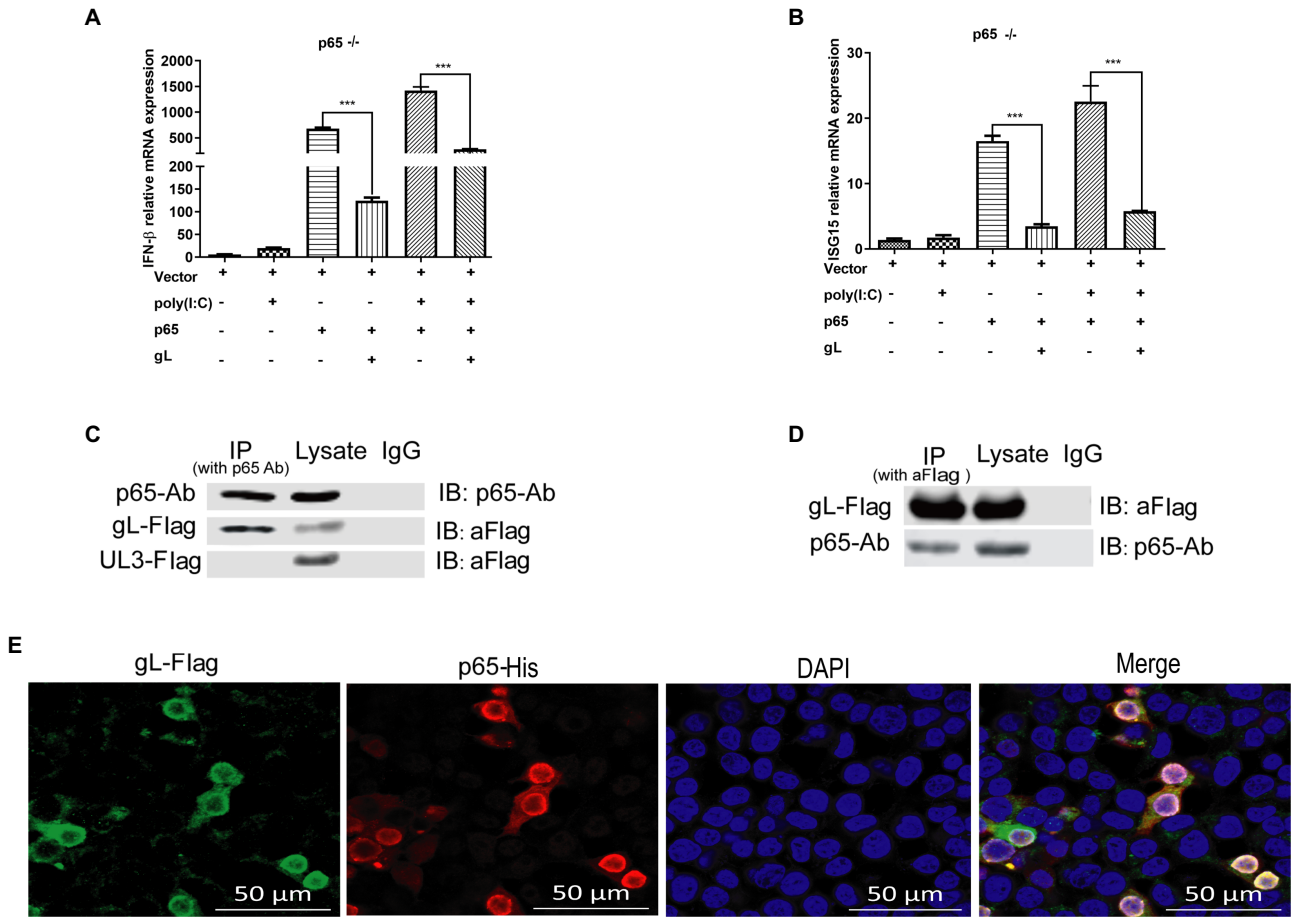
gL interacts with after poly(I:C) induction. **(A,B)** HEK293T cells of p65 knock out (p65^−/−^ cells) were cotransfected with pcDNA3.1-gL (400 ng) and pcDNA3.1- p65(400 ng), or empty plasmid (400 ng) for 32 h. At 32 h posttransfection, cells were harvested and subjected to qRT-PCR analysis. Next, HEK 293 T cells of p65 knock out were transfected as described for the preceding panel for 24 h, and the cells were stimulated with 2 μg/ml poly(I:C) for 8 h, the cells were harvested and subjected to qRT-PCR analysis. **(C,D)** HEK 293 T cells were transfected with pcDNA3.1-gL (400 ng) and pcDNA3.1-UL3 (400 ng, negative control) for 24 h. At 24 h post-transfection, the cells were harvested for IP assay. Lysates were immunoprecipitated (IP) with p65 Ab and immunoblotted (IB) with p65 Ab or anti-Flag. Lysates were IP with anti-Flag, and then IB with p65 Ab or anti-Flag. **(E)** HEK 293 T cells were transfected with gL-Flag and p65 His for 24 h, the cells were immunostained for Flag and His, and then be imaged by confocal microscopy. The data represent results from one of the triplicate experiments. Error bars represent the standard deviation of three independent experiments. Statistical analysis was performed using Student’s *t*-test with the GraphPad Prism 8.0 software.

The data mentioned above suggested that gL mediated regulation of p-p65 nuclear translocation after the IκBα degradation. The result led us to hypothesize that gL might act directly on p65. To explore this hypothesis, we used the clustered regularly interspaced short palindromic repeats (CRISPR)-Cas9 method to knockout *RELA* (p65) in HEK 293 T cells and then verified the effects of p65 and gL coexpression on the mRNA IFN-β and ISG15. We found that IFN-β and ISG15 transcript was significantly downregulated when p65 and gL were coexpressed on the p65 knockout HEK 293 T cells, but only p65 or gL transfection not (*p* < 0.05; Figures 7A,B). Similar results are found while the HEK 293 T cells (p65^−/−^) were cotransfected with p65 and gL and added the poly(I:C) stimulation (*p* < 0.01; Figures 7A,B). The results verify that gL regulates IFN-β and ISG15 expression via p65.

To further determine the effects of gL on p65, HEK293T cells were transfected with gL-Flag, and co-IP/WB was performed with anti-Flag and anti-p65 antibodies. HSV-1 UL3 encoded protein was applied as a negative control to obviate nonspecific interaction. We found that the gL protein was immunoprecipitated by p65 (Figure 7C), and p65 was coimmunoprecipitated with p65 (Figure 7D). Reciprocal coimmunoprecipitation experiments suggested an interaction between p65 and gL. We performed the fluorescence colocalization microscopy assay to more intuitively indicate the molecule interaction between gL and p65. The data confirmed the colocalization of gL and p65 on the cytoplasm around the nucleus (Figure 7E). These data suggested that gL and p65 interact with each other.

### N terminus of gL bound to the RHD of p65 and impeding the translocation of phosphorylated p65 to the nucleus

**Figure 8:**
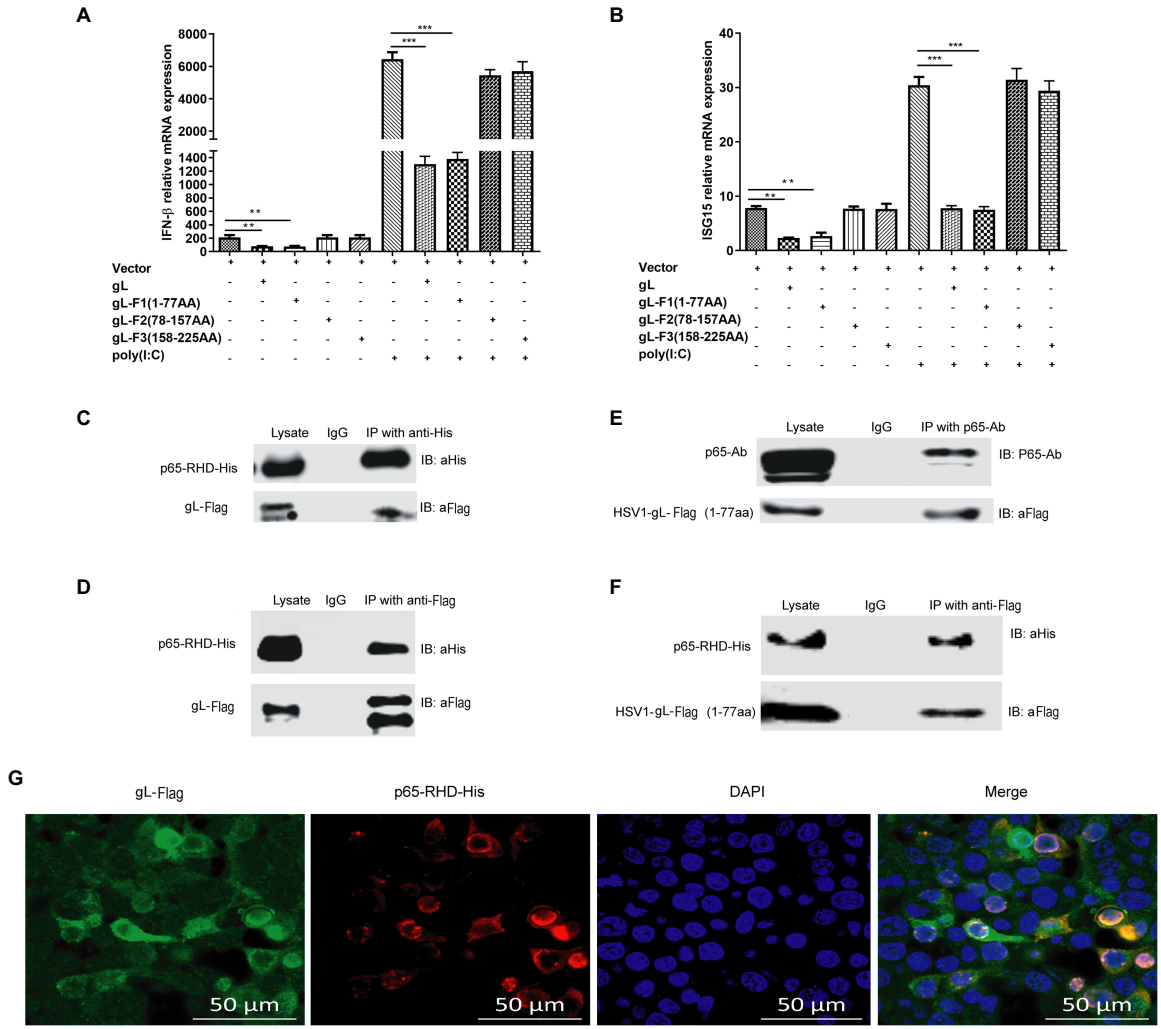
Effects of interactions between gL and p65 on NF-κB signaling. HEK 293 T cells (1 × 10^5^) were transfected with the difference gL-Flag truncated mutants on a relative for 32 h, harvested, and analyzed the mRNA level IFN-β and ISG15. HEK 293 T cells were transfected as described for the preceding panel for 24 h, and then the cells were stimulated with 2 μg/ml poly(I:C) for 8 h; the cells were harvested and subjected to qRT-PCR analysis. **(A)** IFN-β and **(B)** ISG15 levels. **(C-F)** HEK 293T cells (1 × 10^5^) were transfected with pcDNA3.1-gL for 32 h, cells were harvested, and extracted the protein for the IP assay. HEK 293 T cells (1 × 10^5^) were transfected with pcDNA3.1-gL for 24 h, and then the cells were stimulated with 2 μg/ml poly(I:C) for 8 h, and then cells were harvested and extracted the protein for the IP assay. **(C)** Lysates were immunoprecipitated (with anti-His) for His-p65-RHD and immunoblotted with anti-Flag or anti-His. **(D)** Lysates were immunoprecipitated for gL-Flag (with anti-Flag) and immunoblotted with anti-Flag or anti-His. **(E)** p65 coimmunoprecipitated with the 77-amino acid N terminus of gL (1 μg). **(F)** The 77-amino acid N terminus of gL was coimmunoprecipitated with His-tagged p65-RHD. **(G)** HEK293T cells were transfected with the 77-amino acid N terminus of gL-Flag for 24 h, the cells were immunostained for Flag and His and imaged by confocal microscopy. The data represent results from one of the triplicate experiments. Error bars represent the standard deviation of three independent experiments. Statistical analysis was performed using Student’s *t*-test with the GraphPad Prism 8.0 software (**0.001 < *p* < 0.01; ***p < 0.001).

The previous results suggested that gL and p65 interact with each other, consistently overexpressed gL-Flag and His-p65 colocalized in the cytoplasm. To further define the interactional site of gL and p65 during innate sensing, HEK 293 T cells were transfected with three truncation mutant’s gL expression plasmids, and then cells were harvested and subjected to qRT-PCR to analyze IFN-β and ISG15 mRNA. As shown in Figures 8A,B, the gL N terminus (amino acids 1–77) significantly downregulated the transcription of IFN-β and ISG15 (*p* < 0.05), while amino acids 78–225 did not significantly affect IFN-β and ISG15 mRNA levels (Figures 8A,B). Similarly, after poly(I:C) stimulation, the gL N terminus reduced the transcription of IFN-β and ISG15 markedly (*p* < 0.01; Figures 8A,B). The results indicate that the N terminal portion of gL is important for the regulation of NF-κB signaling.

To further investigate the interaction between the N terminal portion of gL and p65-RHD, Coimmunoprecipitation experiments using His and Flag-tagged fragments of p65 and gL, respectively. As expected, gL-Flag coimmunoprecipitated with His-p65 RHD (Figure 8C) and His-p65-RHD coimmunoprecipitated with gL-Flag (Figure 8D). p65 coimmunoprecipitated with the 77-amino acid N terminus of gL (Figure 8E), and His-tagged p65-RHD coimmunoprecipitated with the gL N-terminus (Figure 8F).

To further investigate the interaction between N terminus of gL and p65-RHD, we performed the fluorescence colocalization microscopy experiment using His-tagged p65-RHD, and it also colocalized with Flag-tagged the N terminal portion of gL in the cytoplasm (Figure 8G). Altogether, these results demonstrate that gL inhibits p65 translocation into the nucleus by binding its RHD through its N-terminal region, decreasing IFN production and hindering the host’s resistance to HSV-1.

## Discussion

NF-κB is a critical transcription factor regulating innate immune responses to viral invasion (Medzhitov, 1997; Seth et al., 2005; Le Negrate, 2011). Therefore, for a virus to survive and achieve latency in the host, it must acquire strategies to interfere with NF-κB activity and escape these immune responses (Gregory, 2004). Over the past decade, several studies have demonstrated that HSV-1 invades host cells with the help of the envelope glycoproteins gB, gD, and gH/gL20-28 and then releases various viral proteins to escape the innate immune response (Cai et al., 1988; Chowdhury et al., 2012). These proteins include UL24 (Zhang et al., 2013a,b), large tegument protein UL36 (Zheng and Su, 2017), UL42 (Zhang et al., 2013a,b), US3 (Wang J. P. et al., 2013; Wang S. et al., 2013), ICP0 (Orvedahl et al., 2007), and transactivating tegument protein VP16 (Triezenberg et al., 1988; Huang et al., 2018), all of which have been shown to interact with IRF3 and mediate NF-κB activation, leading to the downregulation of IFN-β production. Other viral proteins, including tegument protein VP22 (Deschamps and Kalamvoki, 2017), tegument protein VP11/12 (Heldwein and Krummenacher, 2008), and infected cell protein ICP27 (Xing et al., 2012), inhibit IFN-β production by targeting stimulator of interferon response cGAMP interactor 1 and TBK1. However, the antiviral immunity-regulating activity of gL remains to be defined. Our results indicate that gL markedly impedes p65 nuclear translocation through direct binding, downregulating the transcription of IFN-β and ISGs to escape host antiviral responses.

Previous studies have indicated that gL binds gH to form a gH/gL heterodimer, promoting HSV-1 envelope membrane fusion with the cell membrane, leading to viral entry (Fuller and Lee, 1992; Peng et al., 1998; Rasmussen et al., 2007; Hayden and Ghosh, 2011; Chowdhury et al., 2012; Gianni et al., 2015; Lau and Crump, 2015; Westra et al., 2000). Integrins compete with gL for the same binding site on gH, and binding to integrin avβ6 promotes gH/gL dissociation (Hayden and Ghosh, 2011; Gianni et al., 2015). Thereby, gL is critical for HSV-1 entry into cells and is also a functional inhibitor of gH, maintaining it in an inactive form until receptor-bound gD and integrins signal otherwise (Gianni et al., 2015). However, no functions have been reported for gL outside the heterodimer. Here, we demonstrate that gL overexpression suppresses the transcription of IFN-β and ISG15. IFN-β is produced upon immune activation in many cell types, including lymphocytes, and has various antiviral signaling functions. We examined the effects of gL on the transcription and translation of antiviral regulatory factors in the NF-κB and IRF3/IRF7 pathways, including receptors and adaptor proteins such as MAVS, RIG-I, IKKs, IκBα, TBK1, INKIT, and p50/p65. Overexpression of gL did not regulate NF-κB pathway component levels nor p65 phosphorylation but instead suppressed its nuclear translocation. On the contrary, the nuclear translocation of p65 was inactivated when gL was disabled by loss-of-function siRNA. In its inactivated state, NF-κB is bound to its inhibitor, IκBα. Upon immune stimulation, IκBα dissociates from NF-κB and is ubiquitinated by IKK, and degraded (Le Negrate, 2011). INKIT has been implicated in the recruitment and phosphorylation of p65 (Huang et al., 1997); however, our data do not support a functional role for INKIT in the gL-mediated regulation of p65. Instead, gL seems to interact directly with p65, inhibiting its nuclear translocation. These data suggest that gL is involved in viral entry into host cells and regulates NF-κB activation.

NF-κB signaling requires that the p50/p65 dimer enter the nucleus, thus blocking the pathway by retaining the dimer in the cytoplasm is an important strategy for viral escape (Zhou et al., 2013). Multiple HSV-1 proteins, including US3, UL24, UL42, and ICP0, can bind p50 or p65 and prevent their nuclear translocation (Orvedahl et al., 2007; Wang J. P. et al., 2013; Wang S. et al., 2013; Zhang et al., 2013a,b; Xu et al., 2017). This study demonstrates that gL binds to the RHD of p65, thus inhibiting its nuclear translocation and downregulating IFN-β and ISG production.

Previously studies have demonstrated that HSV-1 gL residues 23–41,162–224 (including minus-strand residues 197–203) do not affect gH/gL trafficking and function (Davison and Scott, 1986). Therefore, we focused our analysis on the HSV-1 gL N terminus and found that it inhibited NF-κB activation by binding the p65 RHD, blocking its NLS, and inhibiting its nuclear translocation. However, the structural details of how gL binds the RHD of p65 will require further study.

As gL is necessary for membrane fusion and viral entry into host cells (Davison and Scott, 1986; Ligas and Johnson, 1988; Roop et al., 1993; Connolly et al., 2011; Rogalin and Heldwein, 2011; Wang et al., 2016), it is conceivable that defects in gL would impede viral cell entry and decrease the cellular viral titer (Gilles, 1974). To retain gL’s immune function without affecting viral entry, the gL function of HSV-1 must be inactivated by specific targeting after viral infection. To answer this question, cells were infected with wild type and gL-depleted HSV-1. Depletion of gL significantly increased the nuclear translocation of p65 without affecting HSV-1 replication (Supplementary Figure 3). Thus, it is plausible that HSV-1 efficiently evades host antiviral responses by targeting p65.

In summary, we have demonstrated that the HSV-1 glycoprotein gL inhibits NF-κB pathway-mediated antiviral activity by binding the p65 RHD, blocking its NLS, and inhibiting its nuclear translocation, revealing a novel strategy by which HSV-1 evades host innate immunity. These findings broaden our understanding of the mechanisms used by HSV-1 to evade host antiviral responses.

## Data availability statement

The original contributions presented in the study are included in the article/Supplementary material, further inquiries can be directed to the corresponding authors.

## Author contributions

ZL: conceptualization, methodology, software, investigation, formal analysis, writing—original draft, funding acquisition, and writing—review and editing. ZFe: data curation and writing—original draft. ZFa: visualization and investigation. JC: resources and supervision. WC: software and validation. WL: visualization and writing—review and editing. QC: conceptualization, resources, and supervision. All authors contributed to the article and approved the submitted version.

## Conflict of interest

The authors declare that the research was conducted in the absence of any commercial or financial relationships that could be construed as a potential conflict of interest.

## Publisher’s note

All claims expressed in this article are solely those of the authors and do not necessarily represent those of their affiliated organizations, or those of the publisher, the editors and the reviewers. Any product that may be evaluated in this article, or claim that may be made by its manufacturer, is not guaranteed or endorsed by the publisher.

## References

[ref1] Benetti L. Munger J. Roizman B. (2003). The herpes simplex virus 1 US3 protein kinase blocks caspase-dependent double cleavage and activation of the Proapoptotic protein BAD. J. Virol. 77, 6567–6573. doi: 10.1128/jvi.77.11.6567-6573.2003, PMID: 12743316 PMC155029

[ref2] Cai W. H. Gu B. Person S. (1988). Role of glycoprotein B of herpes simplex virus type 1 in viral entry and cell fusion. J. Virol. 62, 2596–2604. doi: 10.1128/JVI.62.8.2596-2604, PMID: 2839688 PMC253689

[ref3] Chowdhury S. Naderi M. Chouljenko V. N. Walker J. D. Kousoulas K. G. (2012). Amino acid differences in glycoproteins B (gB), C (gC), H (gH) and L(gL) are associated with enhanced herpes simplex virus type-1 (McKrae) entry via the paired immunoglobulin-like type-2 receptor. Virol. J. 9:112. doi: 10.1186/1743-422X-9-112, PMID: 22695228 PMC3402990

[ref4] Connolly S. A. Jackson J. O. Jardetzky T. S. Longnecker R. (2011). Fusing structure and function: a structural view of the herpesvirus entry machinery. Nat. Rev. Microbiol. 9, 369–381. doi: 10.1038/nrmicro2548, PMID: 21478902 PMC3242325

[ref5] Davison A. J. Scott J. E. (1986). DNA sequence of the major capsid protein gene of herpes simplex virus type 1. J. Gen. Virol. 67, 2279–2286. doi: 10.1099/0022-1317-67-10-2279 3020164

[ref6] Davis-Poynter N. Bell S. Minson T. Browne H. (1994). Analysis of the contributions of herpes simplex virus type I membrane proteins to the induction of cell-cell fusion. J. Virol. 68, 7586–7590. doi: 10.1128/JVI.68.11.7586-7590, PMID: 7933147 PMC237207

[ref7] Deschamps T. Kalamvoki M. (2017). Evasion of the STING DNA sensing pathway by the VP11/12 of herpes simplex virus type 1. J. Virol. 91, e00535–e00517. doi: 10.1128/JVI.00535-17, PMID: 28592536 PMC5533902

[ref8] Dong X. Guan J. Zheng C. Zheng X. (2017). The herpes simplex virus 1 UL36USP Deubiquitinase suppresses DNA repair in host cells via Deubiquitination of proliferating cell nuclear antigen. J. Biol. Chem. 292, 8472–8483. doi: 10.1074/jbc.M117.778076, PMID: 28348081 PMC5437251

[ref9] Fuller A. Lee W. (1992). Herpes simplex virus type 1 entry through a cascade of virus-cell interactions requires different roles of gD and gH in penetration. J. Virol. 66, 5002–5012. doi: 10.1128/JVI.66.8.5002-5012.1992, PMID: 1321283 PMC241354

[ref10] Gianni T. Massaro R. Campadelli-Fiume G. (2015). Dissociation of HSV gL from gH by αvβ 6- or αvβ8-integrin promotes gH activation and virus entry. Proc. Natl. Acad. Sci. U. S. A. 112, E3901–E3910. doi: 10.1073/pnas.1506846112, PMID: 26157134 PMC4517248

[ref11] Gilles H. J. (1974). Calculation of the index of acute toxicity by the method of linear regression. Comparison with the method of “Karber and Behrens”. Eur. J. Toxicol. Environ. Hyg. J. Eur. Toxicol. 7, 77–84. PMID: 4408718

[ref12] Gregory D. (2004). Efficient replication by herpes simplex virus type 1 involves activation of the IkappaB kinase-IkappaB-p65 pathway. J. Virol. 78, 13582–13590. doi: 10.1128/JVI.78.24.13582-13590.2004, PMID: 15564469 PMC533927

[ref13] Hayden M. S. Ghosh S. (2011). NF-κB in immunobiology. Cell Res. 21, 223–244. doi: 10.1038/cr.2011.13, PMID: 21243012 PMC3193440

[ref14] Heldwein E. E. Krummenacher C. (2008). Entry of herpesviruses into mammalian cells. Cell. Mol. Life Sci. 65, 1653–1668. doi: 10.1007/s00018-008-7570-z 18351291 PMC11131758

[ref15] Herold B. C. Wudunn D. Soltys N. Spear P. G. (1991). Glycoprotein C of herpes simplex virus type 1 plays a principal role in the adsorption of virus to cells and in infectivity. J. Virol. 65, 1090–11098. doi: 10.1128/JVI.65.3.1090-1098.1991, PMID: 1847438 PMC239874

[ref16] Huang D. Huxford T. Chen Y. Ghosh G. (1997). The role of DNA in the mechanism of NFκB dimer formation: crystal structures of the dimerization domains of the p50 and p65 subunits. Structure 5, 1427–1436. doi: 10.1016/S0969-2126(97)00293-1 9384558

[ref17] Huang J. You H. Su C. Li Y. Chen S. Zheng C. (2018). Herpes simplex virus 1 tegument protein VP22 abrogates cGAS/STING-mediated antiviral innate immunity. J. Virol. 92, e00841–e00818. doi: 10.1128/JVI.00841-18, PMID: 29793952 PMC6052299

[ref18] Jin C. K. Lee S. Y. Sang Y. K. Kim J. K. Ahn J. K. (2008). HSV-1ICP27 suppresses NF-kappa B activity by stabilizing I kappa B alpha. FEBS Lett. 582, 2371–2376. doi: 10.1016/j.febslet.2008.05.044 18539148

[ref19] Kleinman S. Vamvakas E. C. (2010). Assessment of the risk of transfusion-transmitted viral infections. Transfus. Altern. Transfus. Med. 5, 319–325. doi: 10.1111/j.1778-428X.2003.tb00169.x

[ref20] Lau K. Crump C. (2015). HSV-1 gM and the gK/pUL20 complex are important for the localization of gD and gH/L to viral assembly sites. Viruses 7, 915–938. doi: 10.3390/v7030915, PMID: 25746217 PMC4379555

[ref21] Le Negrate G. (2011). Viral interference with innate immunity by preventing NF-κB activity. Cell. Microbiol. 14, 168–181. doi: 10.1111/j.1462-5822.2011.01720, PMID: 22050732

[ref22] Ligas M. Johnson D. (1988). A herpes simplex virus mutant in which glycoprotein D sequences are replaced by beta-galactosidase sequences binds to but is unable to penetrate into cells. J. Virol. 62, 1486–1494. doi: 10.1128/JVI.62.5.1486-1494.1988, PMID: 2833603 PMC253172

[ref23] Lu B. Ren Y. Sun X. Han C. Huang Z. (2017). Induction of INKIT by viral infection negatively regulates antiviral responses through inhibiting phosphorylation of p65 and IRF3. Cell Host Microbe 22, 86–98.e4. doi: 10.1016/j.chom.2017.06.013, PMID: 28704656

[ref24] Maclean C. A. (1998). HSV entry and spread. Methods Mol. 10, 9–18. doi: 10.1385/0-89603-347-3:9 21374219

[ref25] Medzhitov R. (1997). Innate immunity: the virtues of a nonclonal system of recognition. Cells 91, 295–298. doi: 10.1016/s0092-8674(00)80412-2, PMID: 9363937

[ref26] Orvedahl A. Alexander D. Tallóczy Z. Sun Q. Wei Y. Zhang W.. (2007). HSV-1 ICP34.5 confers Neurovirulence by targeting the Beclin 1 autophagy protein. Cell Host Microbe 1, 23–35. doi: 10.1016/j.chom.2006.12.001, PMID: 18005679

[ref27] Peng T. Poncedeleon M. Jiang H. Dubin G. Lubinski J. M. Eisenberg R. J.. (1998). The gH-gL complex of herpes simplex virus (HSV) stimulates neutralizing antibody and protects mice against HSV type 1 challenge. J. Virol. 72, 65–72. doi: 10.1128/JVI.72.1.65-72.1998, PMID: 9420201 PMC109350

[ref28] Rasmussen S. B. Sorensen L. N. Malmgaard L. Ank N. Baines J. D. Chen Z. J.. (2007). Type I interferon production during herpes simplex virus infection is controlled by cell-type-specific viral recognition through toll-like receptor 9, the mitochondrial antiviral signaling protein pathway, and novel recognition systems. J. Virol. 81, 13315–13324. doi: 10.1128/JVI.01167-07, PMID: 17913820 PMC2168887

[ref29] Rogalin H. B. Heldwein E. E. (2011). Characterization of vesicular stomatitis virus Pseudotypes bearing essential entry glycoproteins gB, gD, gH, and gL of herpes simplex virus. J. Virol. 90, 10321–10328. doi: 10.1128/JVI.01714-16, PMID: 27605677 PMC5105666

[ref30] Roop C. Hutchinson L. Johnson D. C. (1993). A mutant herpes simplex virus type 1 unable to express glycoprotein L cannot enter cells, and its particles lack glycoprotein H. J. Virol. 67, 2285–2297. doi: 10.1128/JVI.67.4.2285-2297, PMID: 8383241 PMC240370

[ref31] Samady L. Costigliola E. Mac Cormac L. McGrath Y. Cleverley S. Lilley C. E.. (2003). Deletion of the Virion host shutoff protein (vhs) from herpes simplex virus (HSV) relieves the viral block to dendritic cell activation: potential of vhs- HSV vectors for dendritic cell-mediated immunotherapy. J. Virol. 77, 3768–3776. doi: 10.1128/jvi.77.6.3768-3776, PMID: 12610151 PMC149543

[ref32] Seth R. B. Sun L. Ea C. K. Chen Z. J. (2005). Identification and characterization of MAVS, a mitochondrial antiviral signaling protein that activates NF-kappaB and IRF 3. Cells 122, 669–682. doi: 10.1016/j.cell.2005.08.012, PMID: 16125763

[ref33] Su C. Zhang J. Zheng C. (2015). Herpes simplex virus 1 UL41 protein abrogates the antiviral activity of hZAP by degrading its mRNA. Virol. J. 12:203. doi: 10.1186/s12985-015-0433-y, PMID: 26625984 PMC4666169

[ref34] Triezenberg S. J. LaMarco K. L. McKnight S. L. (1988). Evidence of DNA: protein interactions that mediate HSV-1 immediate early gene activation by VP16. Genes Dev. 2, 730–742. doi: 10.1101/gad.2.6.730, PMID: 2843426

[ref35] Wang J. P. Bowen G. N. Zhou S. Cerny A. Zacharia A. Knipe D. M.. (2013). Role of specific innate immune responses in herpes simplex virus infection of the central nervous system. J. Virol. 86, 2273–2281. doi: 10.1128/JVI.06010-11, PMID: 22171256 PMC3302371

[ref36] Wang Y. Huang L.-P. Du W.-J. Wei Y.-W. Wu H.-L. Feng L.. (2016). Targeting the pseudorabies virus DNA polymerase processivity factor UL42 by RNA interference efficiently inhibits viral replication. Antivir. Res. 132, 219–224. doi: 10.1016/j.antiviral.2016.06.010, PMID: 27387827

[ref37] Wang S. Wang K. Lin R. Zheng C. (2013). Herpes simplex virus 1 serine/threonine kinase US3 Hyperphosphorylates IRF3 and inhibits Beta interferon production. J. Virol. 87, 12814–12827. doi: 10.1128/JVI.02355-13, PMID: 24049179 PMC3838156

[ref38] Westra D. F. Van Kooij A. Welling G. Scheffer A. J. The T. H. Wellingwester S.. (2000). Natural infection with herpes simplex virus type 1 (HSV-1) induces humoral and T cell responses to the HSV-1 glycoprotein H:L complex. J. Gen. Virol. 81, 2011–2015. doi: 10.1099/0022-1317-81-8-2011 10900040

[ref39] Wylie K. M. Weinstock G. M. Storch G. A. (2012). Emerging view of the human virome. Transl. Res. 160, 283–290. doi: 10.1016/j.trsl.2012.03.006, PMID: 22683423 PMC3701101

[ref40] Xing J. Wang S. Lin R. Mossman K. L. Zheng C. (2012). Herpes simplex virus 1 tegument protein US11 Downmodulates the RLR signaling pathway via direct interaction with RIG-I and MDA-5. J. Virol. 86, 3528–3540. doi: 10.1128/JVI.06713-11, PMID: 22301138 PMC3302539

[ref41] Xu H. Su C. Pearson A. Mody C. H. Zheng C. Sandri-Goldin R. M. (2017). Herpes simplex virus 1 UL24 abrogates the DNA sensing signal pathway by inhibiting NF-κB activation. J. Virol. 91, e00025–e00017. doi: 10.1128/JVI.00025-17 28100608 PMC5355614

[ref42] Yuan H. You J. You H. Zheng C. (2018). Herpes simplex virus 1 UL36USP antagonizes type I interferon-mediated antiviral. Innate Immun. 92, e01161–e01118. doi: 10.1128/JVI.01161-18 PMC6146802 29997210

[ref43] Zhang D. Su C. Zheng C. (2016). Herpes simplex virus 1 serine protease VP24 blocks the DNA-sensing signal pathway by abrogating activation of interferon regulatory factor. J.Virol. 90, 5824–5829. doi: 10.1128/JVI.00186-16, PMID: 27076640 PMC4886774

[ref44] Zhang J. Wang K. Wang S. Zheng C. (2013a). Herpes simplex virus 1 E3 ubiquitin ligase ICP0 protein inhibits tumor necrosis factor alpha-induced NF-κB activation by interacting with p65/RelA and p50/NF-κB1. J. Virol. 87, 12935–12948. doi: 10.1128/JVI.01952-13, PMID: 24067962 PMC3838126

[ref45] Zhang J. Wang S. Wang K. Zheng C. (2013b). Herpes simplex virus 1 DNA polymerase processivity factor UL42 inhibits TNF-α-induced NF-κB activation by interacting with p65/RelA and p50/NF-κB1. Med. Microbiol. Immunol. 202, 313–325. doi: 10.1007/s00430-013-0295-0, PMID: 23636254

[ref46] Zheng C. Su C. (2017). Herpes simplex virus 1 infection dampens the immediate early antiviral innate immunity signaling from peroxisomes by tegument protein VP16. Virol. J. 14:35. doi: 10.1186/s12985-017-0709-5, PMID: 28222744 PMC5320731

[ref47] Zhou W. Chen F. Klyachkin Y. Sham Y. Geraghty R. (2013). Mutations in The amino terminus of herpes simplex virus type 1 gL can reduce cell-cell fusion without affecting gH/gL trafficking. J. Virol. 88, 739–744. doi: 10.1128/JVI.02383-13, PMID: 24155377 PMC3911704

