# Supplementary material for: Herpes simplex virus type I glycoprotein L evades host antiviral innate immunity by abrogating the nuclear translocation of phosphorylated NF-κB sub-unit p65
Source: Front Microbiol. 2023 May 9;14:1178249. doi: 10.3389/fmicb.2023.1178249 (PMC10203706; doi:10.3389/fmicb.2023.1178249)
Supplement: Supplementary file 2 [file Data_Sheet_2.docx]

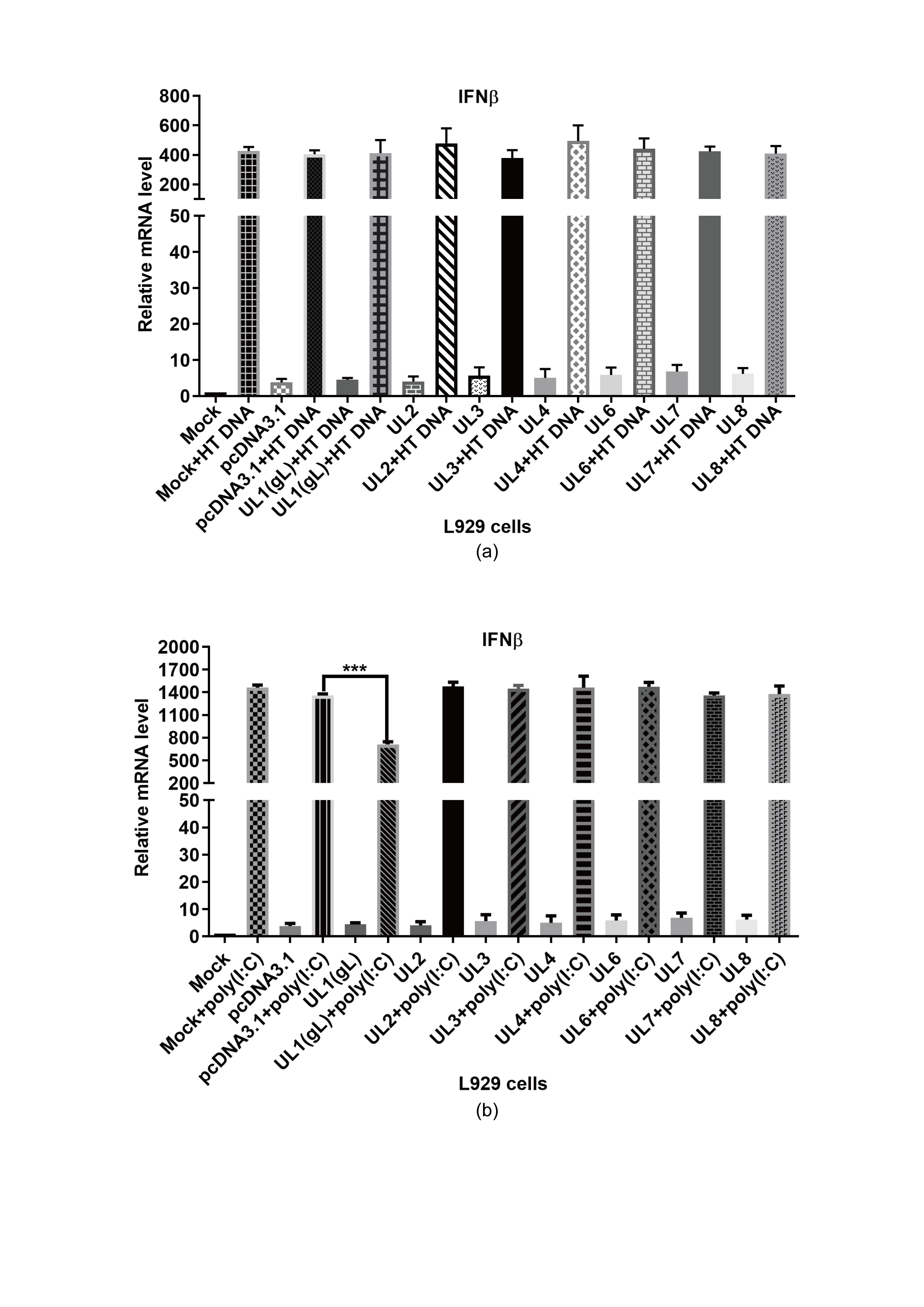
**Figure 1- Over-expr****esed** ***UL、UL2、UL3、UL4、UL6、UL7* and *UL8* of HSV-1 mediated the** **transcription of IFN β**

(a) Over-expressed HSV-1 *UL1,UL2,UL3,UL4,UL6,UL7,UL8* gene mediated the transcription of IFN β under the HT DNA. (b) Over-expressed HSV-1 *UL1,UL2,UL3,UL4,UL6,UL7,UL8* gene mediated the transcription of IFN β under the poly (I:C).
