# Supplementary material for: Herpes simplex virus type I glycoprotein L evades host antiviral innate immunity by abrogating the nuclear translocation of phosphorylated NF-κB sub-unit p65
Source: Front Microbiol. 2023 May 9;14:1178249. doi: 10.3389/fmicb.2023.1178249 (PMC10203706; doi:10.3389/fmicb.2023.1178249)
Supplement: Supplementary file 3 [file Data_Sheet_3.docx]

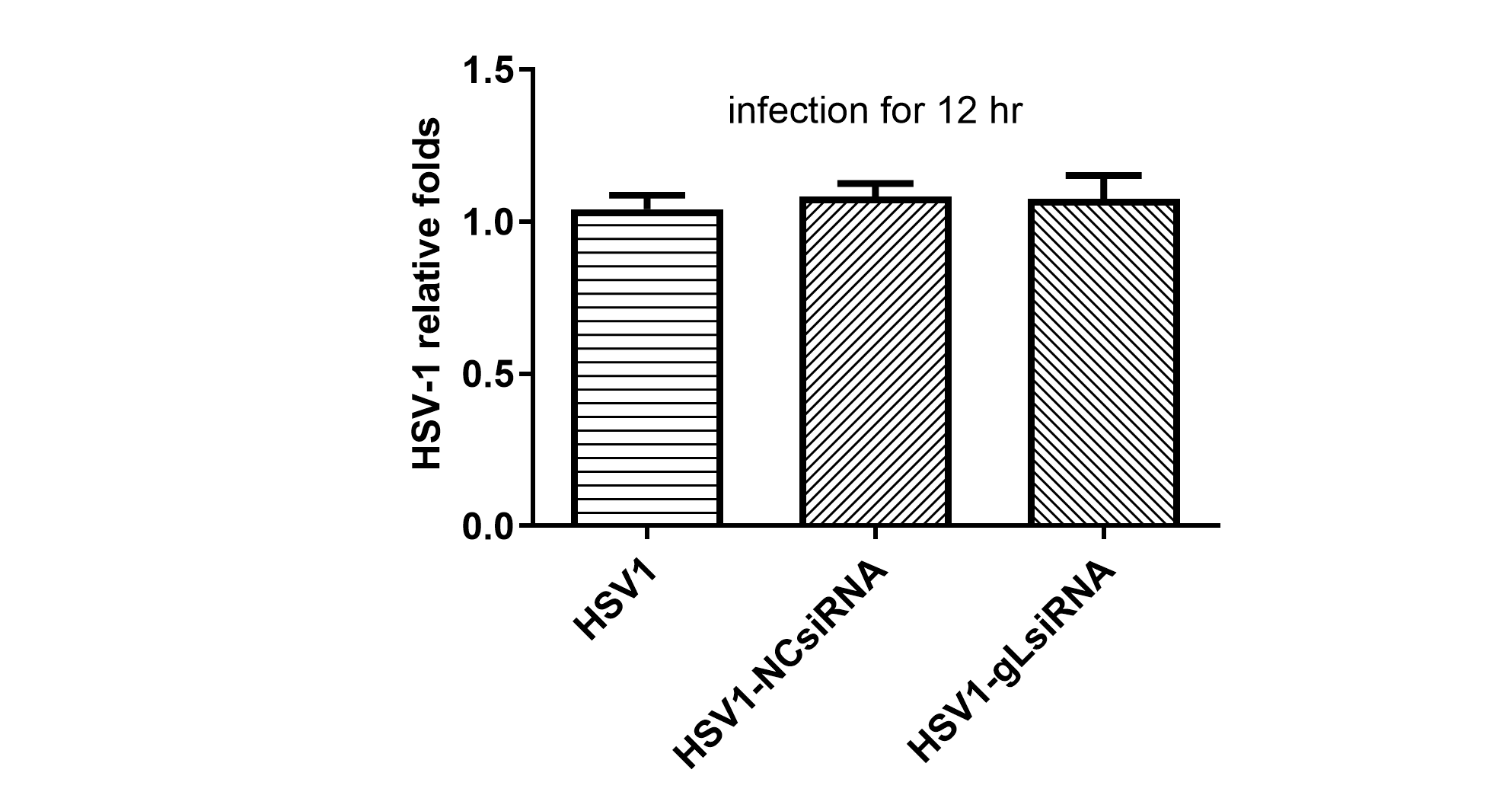


**Figure 1 HSV-1 relative copies**

HEK 293T cells were infected with HSV1, HSV-1+NCsiRNA and HSV-1 +glsiRNA for 12 hr, the copies of HSV-1 not significantly at difference group.
