# Supplementary figures and images for: Herpes simplex virus type I glycoprotein L evades host antiviral innate immunity by abrogating the nuclear translocation of phosphorylated NF-κB sub-unit p65
Source: Front Microbiol. 2023 May 9;14:1178249. doi: 10.3389/fmicb.2023.1178249 (PMC10203706; doi:10.3389/fmicb.2023.1178249)

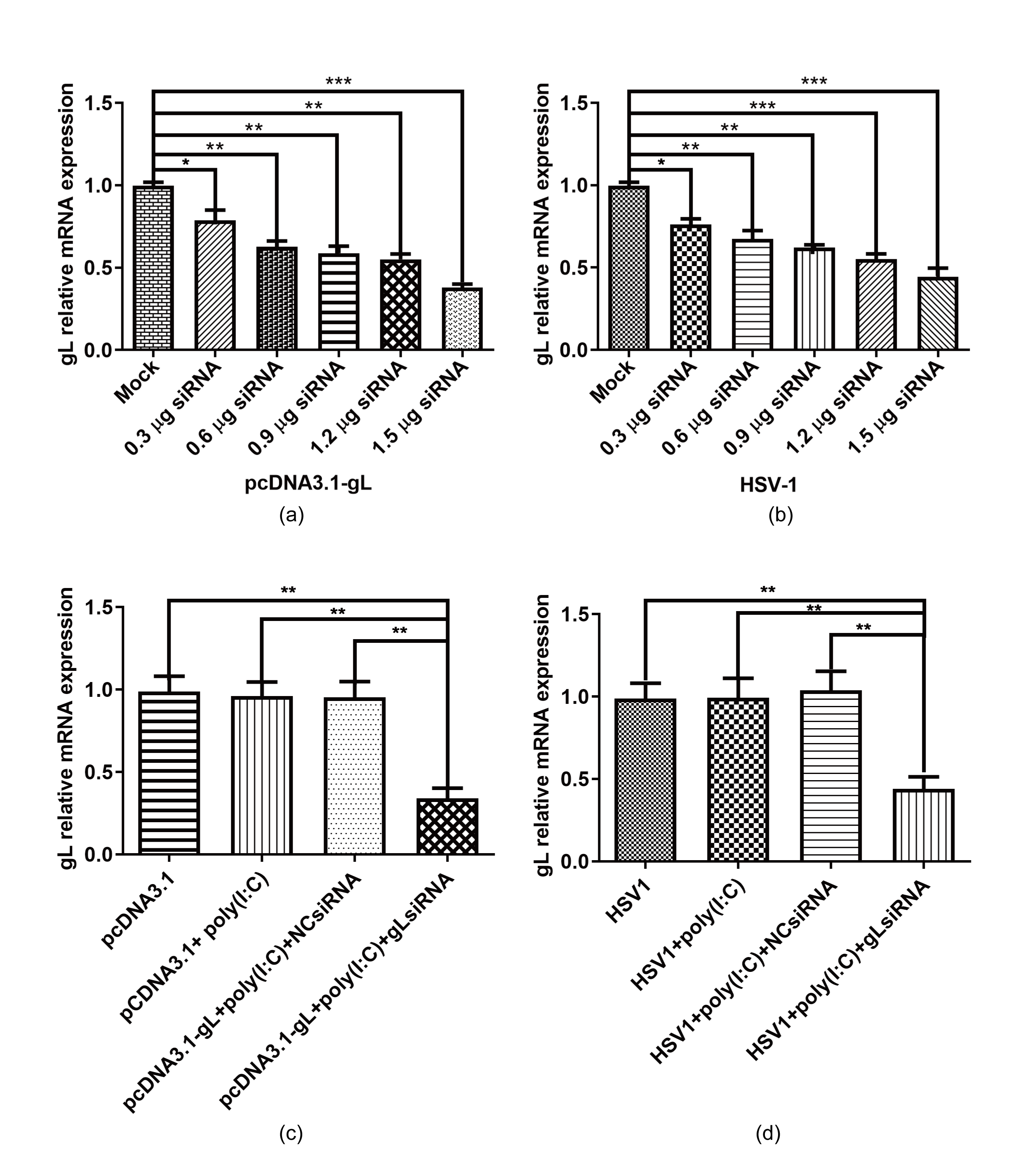

Supplement: Supplementary file 4 [file Image_1.TIF]
